# Shedding light on melanins within *in situ* human eye melanocytes using 2-photon microscopy profiling techniques

**DOI:** 10.1038/s41598-019-54871-y

**Published:** 2019-12-09

**Authors:** Ephrem Sitiwin, Michele C. Madigan, Enrico Gratton, Svetlana Cherepanoff, Robert Max Conway, Renee Whan, Alexander Macmillan

**Affiliations:** 1 School of Optometry and Vision Science, University of New South Wales, Kensington, NSW 2052 Australia; 2 Biomedical Imaging Facility, University of New South Wales, Kensington, NSW 2052 Australia; 3 Laboratory for Fluorescence Dynamics, Department of Biomedical Engineering, University of California Irvine, California, USA; 4 Save Sight Institute, University of Sydney, NSW 2000 Sydney, Australia; 5 St Vincent’s Hospital Sydney, Darlinghurst, NSW 2010 Australia

**Keywords:** Biological fluorescence, Cancer imaging, Eye cancer, Multiphoton microscopy

## Abstract

Choroidal melanocytes (HCMs) are melanin-producing cells in the vascular uvea of the human eye (iris, ciliary body and choroid). These cranial neural crest-derived cells migrate to populate a mesodermal microenvironment, and display cellular functions and extracellular interactions that are biologically distinct to skin melanocytes. HCMs (and melanins) are important in normal human eye physiology with roles including photoprotection, regulation of oxidative damage and immune responses. To extend knowledge of cytoplasmic melanins and melanosomes in label-free HCMs, a non-invasive ‘fit-free’ approach, combining 2-photon excitation fluorescence lifetimes and emission spectral imaging with phasor plot segmentation was applied. Intracellular melanin-mapped FLIM phasors showed a linear distribution indicating that HCM melanins are a ratio of two fluorophores, eumelanin and pheomelanin. A quantitative histogram of HCM melanins was generated by identifying the image pixel fraction contributed by phasor clusters mapped to varying eumelanin/pheomelanin ratio. Eumelanin-enriched dark HCM regions mapped to phasors with shorter lifetimes and longer spectral emission (580–625 nm) and pheomelanin-enriched lighter pigmented HCM regions mapped to phasors with longer lifetimes and shorter spectral emission (550–585 nm). Overall, we demonstrated that these methods can identify and quantitatively profile the heterogeneous eumelanins/pheomelanins within *in situ* HCMs, and visualize melanosome spatial distributions, not previously reported for these cells.

## Introduction

The choroid is a pigmented, richly vascular layer between the neural retina and outer protective sclera of the eye, and the largest component of the uveal tract (choroid, ciliary body and iris). It is essential for supplying oxygen and nutrients to, and removing waste from, the metabolically active adjacent outer retinal layers, the retinal pigment epithelium (RPE) and photoreceptors^1^.

During development, tissues such as the eye, skin, hair, inner ear and brain meninges are populated by immature melanocytes (melanoblasts) that migrate from different regions of the neural crest at different times^2–4^, and display distinct biological characteristics. Adult uveal melanocytes follow a unique developmental pathway (cranial neural crest, pre-migration specification), a mesodermal destination, and distinct tissue distribution, gene expression patterns and physiology when compared to skin melanocytes^5,6^. In the adult human eye, uveal melanocytes are considered integral to normal homeostasis and function, with roles in light absorption, regulation of oxidative stress and roles in immune regulation, angiogenesis and inflammation^7^. Malignant transformation of eye melanocytes can also progress to primary eye (uveal) melanoma^8,9^.

Within melanocytes, the cytoplasmic melanins are synthesized and stored in melanosome organelles^10,11^. The complex melanin biopolymers derived from tyrosine are mixtures of dark (brown to black) eumelanin and light (yellow to red) pheomelanin^12^. Eumelanin, an anti-oxidant, is comprised of indoles with a nitrogen heteroatom, while pheomelanin, a pro-oxidant, is derived from both benzothiazines and benzothiazones with a sulfur heteroatom within the ring structures^13–15^. Skin melanocyte melanins and melanosomes are extensively studied. However, the eumelanin/pheomelanin composition, spatial distributions and intracellular trafficking of melanosomes in (biologically distinct) uveal melanocytes, remains for the most part enigmatic^12,16,17^.

The most common method currently used for quantifying melanins in biological tissues (including eye tissue) is High Performance Liquid Chromatography (HPLC)^17,18^, requiring tissue melanin extraction and chemical degradation. Other techniques such as reflectance microscopy-spectroscopy^19^ and diffuse optical spectroscopy^20^ can be used to study intracellular melanins without cell lysis but do not identify or quantify species of melanins (eumelanins and pheomelanins). Non-linear optical techniques such as pump-probe or transient absorption spectrometry have been successfully applied to visually distinguish the two melanin species in fixed skin tissue^21,22^. Coherent Anti-Stokes Raman Scattering (CARS) microscopy has also been used to directly visualize pheomelanin in mouse skin and hair *in vivo* and in human skin amelanotic melanoma *in situ*^23^. To the best of our knowledge, techniques such as pump-probe, transient absorption spectrometry or CARS have not yet been used to study human eye melanocytes, melanins and/or melanosomes.

To address the above limitations, and to extend our knowledge of eye choroidal melanocyte biology, we applied non-linear 2-photon excitation microscopy (2PM) to non-invasively capture melanin endogenous fluorescence, and to visualize the spatial characteristics and ratios of cytoplasmic melanins in label-free fixed human choroidal melanocytes (HCMs). This allowed effective targeting and excitation of endogenous melanins (and other fluorophores) within full-thickness choroid tissue, with non-destructive deeper tissue scanning and significantly reduced light scattering^24,25^. In the current study, 2PM fluorescence lifetime imaging microscopy (FLIM) and spectral data were acquired. Fluorescence lifetime is defined as the average time the fluorophore remains in the excited state before returning to the ground state^26^. Lifetime and spectral information provide photophysical information with high spatial resolution such as molecular composition and organization, among other properties^27^.

The acquired 2PM FLIM and spectral data were Fourier transformed and analyzed using phasor plot segmentation, a powerful ‘fit-free’ analysis tool that processes unbiased raw data without approximation, requires no *a priori* knowledge of sampled tissues, and yields instantaneous visualization of fluorophore spatial distribution within the image^28,29^. Phasor segmentation was guided by FLIM and spectral phasor profiles from melanin controls and other choroid fluorophores. We thus generated robust quantitative profiles of cytoplasmic melanins (eumelanin/pheomelanin) and melanosomes within label-free heterogeneously pigmented HCMs. As far as we know, this is the first time that this technique has been used to study HCMs *in situ*^30,31^. Our technique may serve as a basis for further exploring the roles of melanins in eye melanocytic lesions. As 2PM imaging techniques evolve for *in vivo* eye tissue examination^31^, these may provide complementary/additional diagnostic tools to assess pigmented eye lesions based on endogenous melanin fluorescence spectral and lifetime signatures. It is also interesting to note that fluorescence lifetime imaging has been applied to study ocular fundus autofluorescence *in vivo*^32,33^.

## Results

### 2PM of HCMs and surrounding human choroidal tissue *in situ*

Label-free and fixed HCMs in culture and *in situ* (choroidal flatmounts and paraffin-embedded sections) were imaged by brightfield and 2PM (Supplementary Note S1). 2-photon excitation was performed at 780 nm, the optimal intracellular melanin excitation (Supplementary Note S2). 2PM images were collected in two channels (500–550 nm and 575–610 nm, with red and green lookup tables applied respectively) that when overlaid, showed colocalizing pixels colored yellow. The fluorescence emission detected from dark pigmented eumelanin enriched HCMs was uniformly green (575–610 nm emission). The light pigmented pheomelanin dominant HCMs showed mostly yellow fluorescence (mainly 500–550 nm emission). The mixed pigmented HCMs, with mixed eumelanin and pheomelanin, showed both green and yellow fluorescence emission.

### Spectral phasor profiling of fluorophore controls

**Figure 1:**
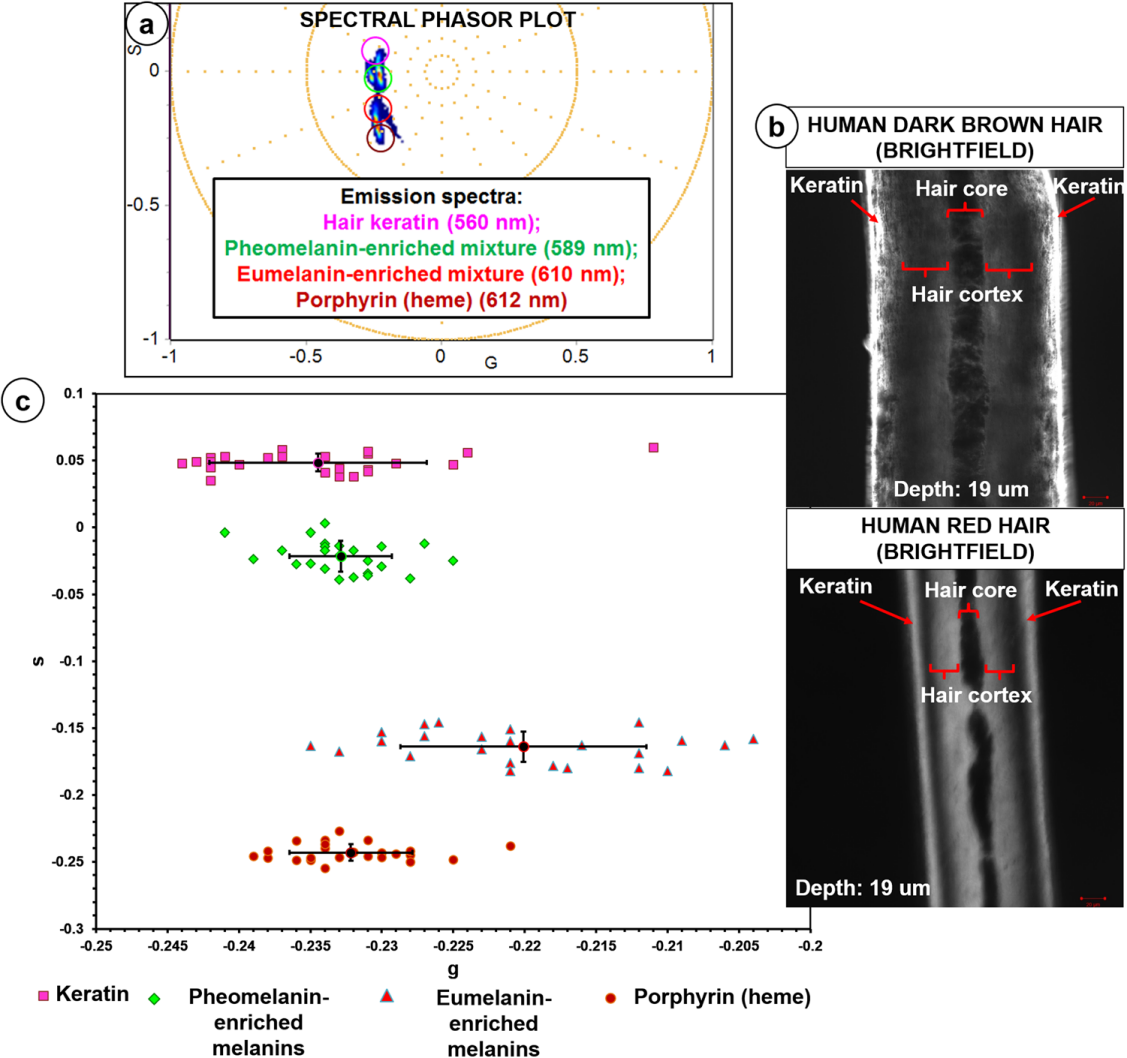
2PM spectral phasor profiling of fluorophore controls. Spectral phasor distribution profiles of various fluorophores used as controls for 2PM spectral phasor segmentations of intracellular melanin and surrounding choroidal components. (**a**) The peak wavelengths of the spectral phasor center of mass related to fluorophores: Hair keratin (560 nm), Dark brown human hair cortex (melanin pigmentation: ‘dark’ to ‘very dark’; eumelanin-enriched; 610 nm), Red human hair cortex (melanin pigmentation: ‘mixed’ to ‘very light’; pheomelanin-enriched; 589 nm), Porphyrin complex (heme) in RBCs (612 nm). (**b**) Human dark brown and red hair cortex were examined at z depth of 19 µm. (**c**) Scatter plot of 24 average points from the spectral phasor distributions corresponding to the following fluorophores (n = 3): hair keratin (K), pheomelanin-enriched red human hair cortex (RH), eumelanin-enriched dark brown human hair cortex (DH) and porphyrin complex (heme) (H) in RBCs. The standard deviation (SD) for each population of average points was generated. The differences, in terms of s values, between the following pairs of average point populations (K versus RH; K versus DH; RH versus DH; H versus RH; H versus DH) were statistically significant (t test, P < 0.05). G = X coordinate of phasor transform (‘real’ unitless phasor component), S = Y coordinate of phasor transform (‘imaginary’ unitless phasor component).

Spectral phasor profiles of various fluorophore controls, including endogenous extracellular matrix (ECM) and porphyrin complex (heme) in red blood cells (RBCs) fluorophores within the human choroid tissue, were obtained at 780 nm (Supplementary Note 1). The fluorescence emission measured from the fluorophore controls displayed overlapping spectra (Fig. 1a, Supplementary Note 1). The peak wavelengths of the spectral phasor center of mass related to fluorophores presented in Fig. 1a were determined: hair keratin (**K**; 560 nm), pheomelanin-enriched melanins in human red hair cortex (**RH**; z = 19 µm; 589 nm), eumelanin-enriched melanins in human dark brown hair cortex (**DH**; z = 19 µm; 610 nm) and porphyrin complex (heme) in RBCs (**H**; 612 nm). The differences, in terms of s values, between pairs of average point populations (**K** versus **RH**; **K** versus **DH**; **RH** versus **DH**; **H** versus **RH**; **H** versus **DH**) were also statistically significant (t test, P < 0.05) (Fig. 1c).

### Spectral phasor profiling of intracellular melanin in cultured HCMs

The image segmentation of the melanin-derived spectral phasor distribution was carefully matched with the subsequent image segmentation of the FLIM phasor distribution by mapping to the same intracellular regions of the sampled HCMs.

**Figure 2:**
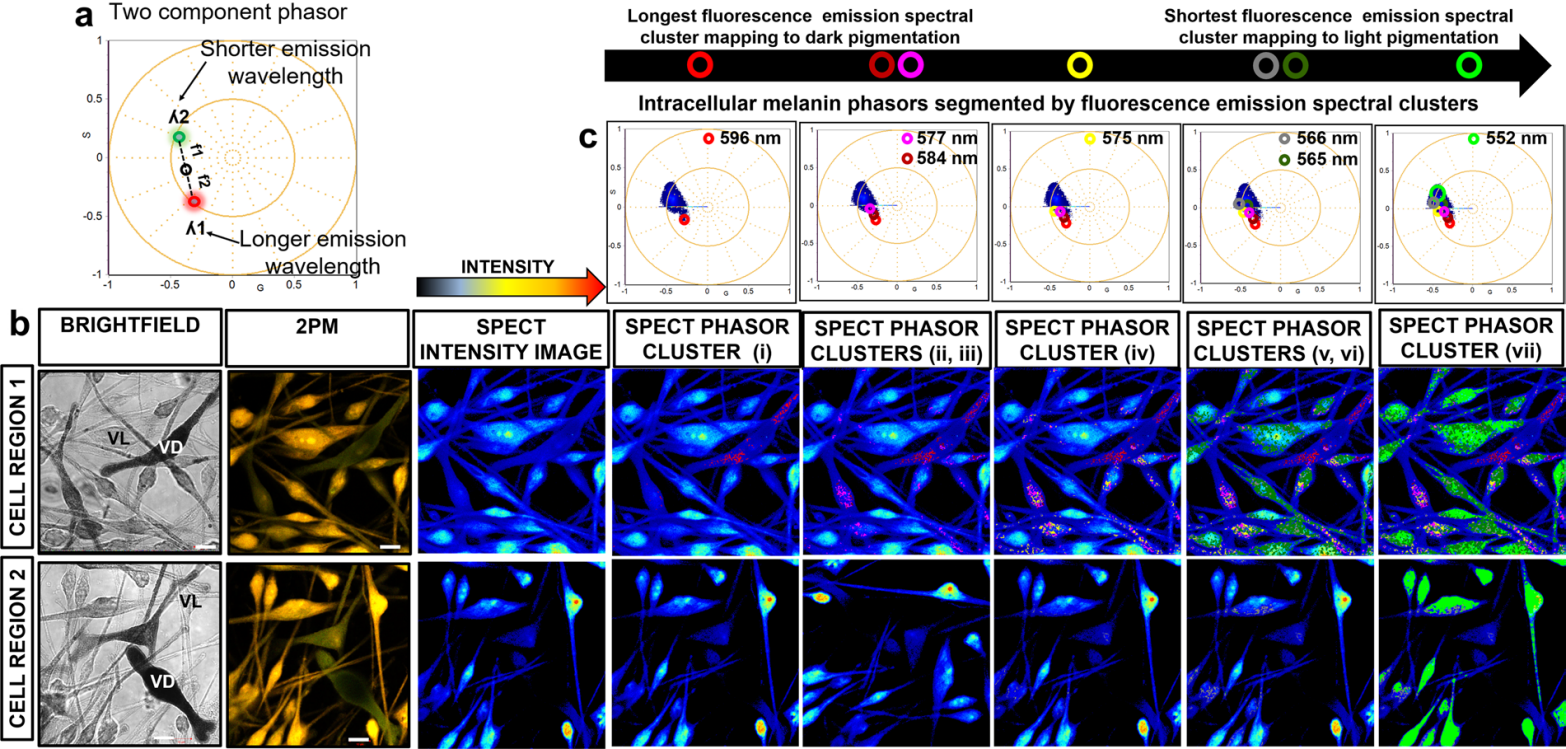
2PM spectral phasor profiling of intracellular melanin in HCM cells. (**a**) Spectral phasors follow rules of vector addition and orthogonality like FLIM phasors. (**b**) Cultured HCMs with heterogeneous pigmentation and morphology were imaged by brightfield and 2PM. (**c**) Segmentation of the melanin spectral phasors was matched with the segmentation of FLIM phasors by mapping to the same regions of the sampled HCMs. Seven spectral phasor clusters mapping to a ratio of eumelanin and pheomelanin were segmented with overlapping fluorescence emission spectra. All spectral emission phasor clusters were also mapped to melanosome-enclosed melanins in both HCM cell bodies and peripheral regions. G = X coordinate of phasor transform (‘real’ unitless phasor component), S = Y coordinate of phasor transform (‘imaginary’ unitless phasor component). Scale bar = 10 μm.

Seven phasor clusters mapping to a eumelanin/pheomelanin ratio were segmented (Fig. 2). Cluster **(i)** mapped to ‘very dark’ intracellular melanin with the fluorescence emission detected at 596 nm. Clusters **(ii)** and **(iii)** mapped to ‘very dark’ to ‘dark’ intracellular melanin with fluorescence emission measured at 584 and 577 nm respectively. Cluster **(iv)** mapped to ‘medium’ pigmented intracellular melanin with fluorescence emission identified at 575 nm. Clusters **(v)** and **(vi)** mapped to the ‘light’ to ‘very light’ intracellular melanin with fluorescence emission detected at 566 and 565 nm. Cluster **(vii)** mapped to ‘very light’ intracellular melanin with fluorescence emission detected at 552 nm. The shift in peak emission identified between dark eumelanin-enriched and light pheomelanin-enriched melanocytes was around 45 nm, greater than a melanin concentration dependent shift (Supplementary Note 2). The segmentation of the spectral phasor distribution from the darker eumelanin-enriched cytoplasm was more difficult than the lighter pheomelanin-enriched cytoplasm because of the poorer signal to noise data from the darker cells. Intracellular melanins in HCMs are synthesized and stored in melanosomes^10,11^, and we observed that the image segmented phasor clusters mapped to melanosomes in HCM cell bodies (perinuclear) and peripheral cell processes. The merged segmented phasor clusters from cultured HCMs and the spectral phasor distributions from the melanin fluorophore controls is shown in Supplementary Figure 2d.

### FLIM phasor profiling of fluorophore controls

**Figure 3:**
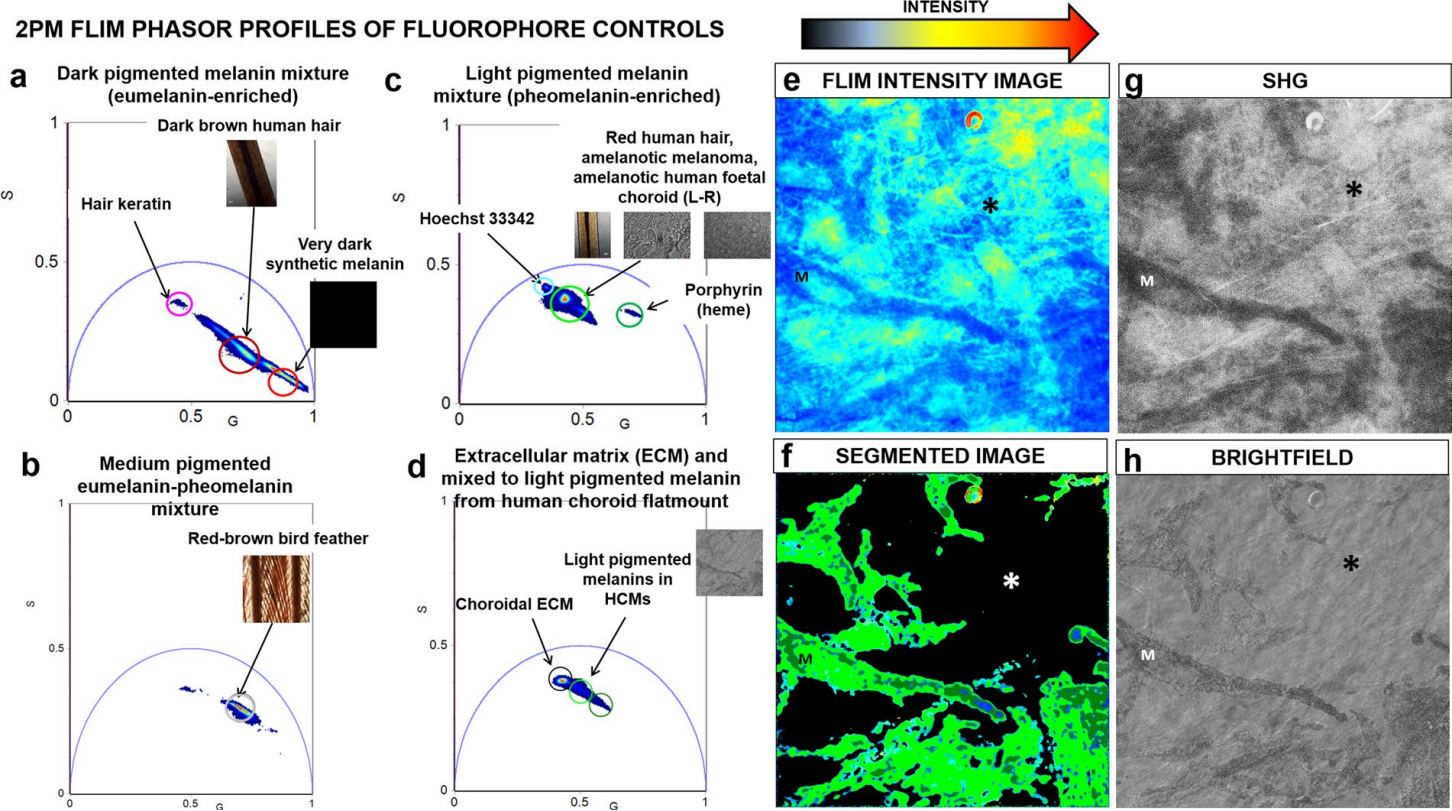
2PM FLIM phasor fingerprints of fluorophore controls. FLIM phasor distribution profiles of various control fluorophores. (**a**) FLIM phasor clusters mapping to synthetic melanin and dark brown human hair cortex (melanin pigmentation: ‘dark’ to ‘very dark’; eumelanin-enriched) with short average fluorescence lifetimes. (**b**) FLIM phasor clusters mapping to red-brown bird feather (melanin pigmentation: ‘medium’; medium mixture of eumelanin and pheomelanin) with medium average fluorescence lifetimes. (**c**) FLIM phasor clusters mapping to red human hair cortex, amelanotic melanoma and lightly pigmented human fetal eye choroid (melanin pigmentation: ‘light’ to ‘very light’; pheomelanin-enriched) with long average fluorescence lifetimes. (**d**) FLIM phasor clusters mapping to choroidal ECM and light pigmented melanins in human choroid flatmount tissue. (**e**) FLIM intensity image of sampled choroid flatmount tissue. (**f**) Segmented FLIM image of light pigmented HCMs and ECM in choroidal tissue. (**g**) The collagen fibers (*) in the ECM surrounding the HCMs were imaged in SHG mode (excitation wavelength: 840 nm, emission range: 420/10 nm). (**h**) Brightfield image of the choroid tissue. M = Melanocytes, * = Collagen fibers, SHG = Second harmonic generation, G = X coordinate of phasor transform (‘real’ unitless phasor component), S = Y coordinate of phasor transform (‘imaginary’ unitless phasor component).

FLIM phasor distribution profiles of various fluorophores were used as controls for choroid components. These were excited at 780 nm and used for subsequent phasor segmentation analysis of HCM-localized melanin and choroid tissue (Fig. 3, Supplementary Note 3). The phasor cluster mapping to choroidal ECM surrounding the HCMs was distinct from the melanins (Fig. 3d). The collagen fibers in the ECM surrounding the HCMs were also verified using second harmonic generation (SHG) imaging (excitation wavelength: 840 nm, emission range: 420/10 nm) (Fig. 3g). The merged phasor plot of all fluorophore controls is presented in Supplementary Note 3.

### FLIM phasor profiling of intracellular melanin in HCMs

Phasor characteristics of HCM intracellular melanins *in vitro* was initially established. FLIM phasor plots derived from ‘very dark’ to ‘very light’ pigmented HCMs was segmented by mapping the phasor clusters to the HCM melanin content within the image.

**Figure 4:**
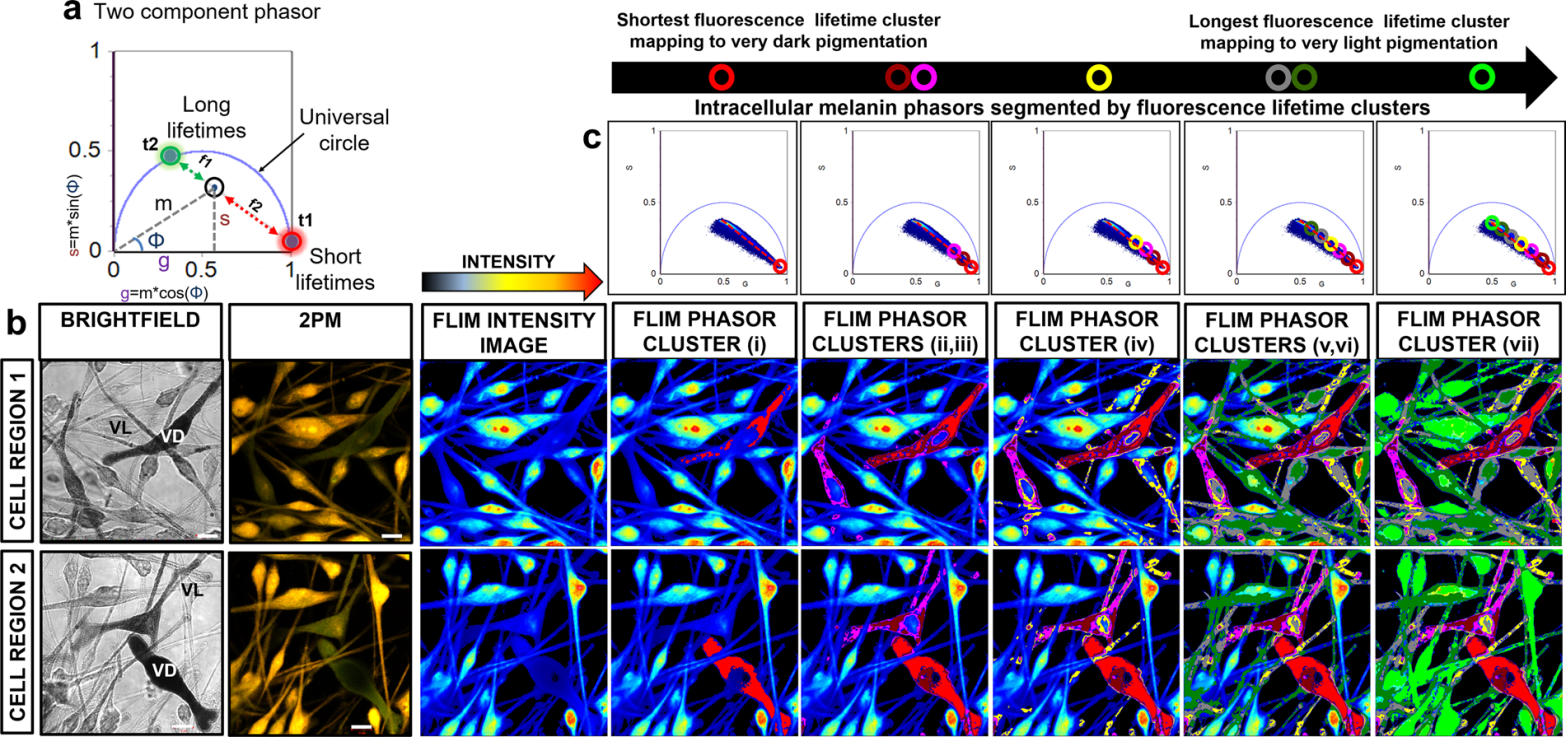
2PM FLIM phasor profiling of intracellular melanin in HCM cells. (**a**) All single fluorophore phasors lie on the universal circle (e.g. t1 & t2; t1 > t2). Multi-component phasors are a linear combination of their fractions. The ratio of the linear combination determines the fraction of the fluorophore species (e.g. f1 & f2). (**b**) Cultured HCMs with heterogeneous pigmentation and morphology were imaged by brightfield and 2PM. (**c**) Seven linearly distributed FLIM phasor clusters were consistently identified post-segmentation, each cluster mapping to a eumelanin/pheomelanin ratio mixture. From shortest to longest average fluorescence lifetimes, the order of the segmented FLIM phasor clusters was: (**i**) < (**ii**) < (**iii**) < (**iv**) < (**v**) < (**vi**) < (**vii**). The image segmented phasor clusters were mapped to melanosomes in cell bodies (perinuclear) and peripheral cell processes of HCMs *in vitro*. G = X coordinate of phasor transform (‘real’ unitless phasor component), S = Y coordinate of phasor transform (‘imaginary’ unitless phasor component), Scale bar = 10 μm.

Seven linearly distributed phasor clusters were identified post-segmentation that consistently showed the spatial distribution of the relative mixtures of eumelanin and pheomelanin across the whole FLIM image of HCMs (Fig. 4c). The phasor cluster identified with the shortest average fluorescence lifetime **(i)** was mapped to intracellular regions of HCMs with the darkest pigmentation. The next two segmented phasor clusters **(ii & iii)** were identified with longer average fluorescence lifetimes than **(i)** and mapped to ‘very dark’- ‘dark’ pigmented cell regions. Cluster (**iv**) was mapped to HCM regions with ‘medium’ level of pigmentation. Clusters **(v)** and **(vi)** were mapped to HCM regions with ‘light’ to ‘very light’ pigmentation. The segmented cluster with the longest average fluorescence lifetime was mapped to ‘very light’ pigmented regions of HCMs (**vii**). The order of the segmented FLIM phasor clusters (shortest to longest average fluorescence lifetimes) was: (**i**) < (**ii**) < (**iii**) < (**iv**) < (**v**) < (**vi**) < (**vii**). As observed for image segmented spectral phasor clusters, the FLIM phasor clusters also mapped to melanosomes within cell bodies (perinuclear) and peripheral cell processes. The merged segmented phasor clusters from cultured HCMs and the FLIM phasor distributions from the melanin fluorophore controls is shown in Supplementary Note 4.

**Figure 5:**
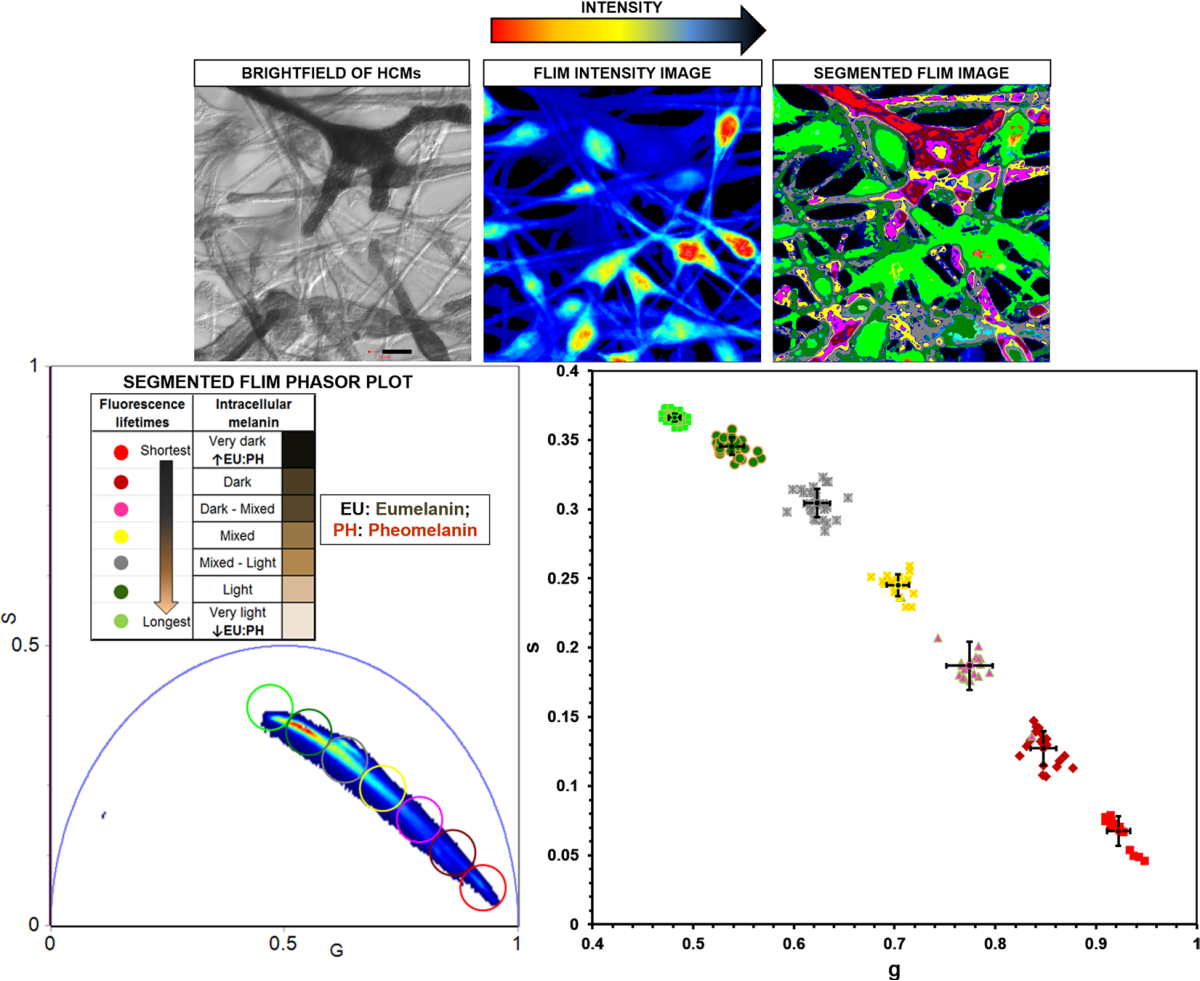
Statistically significant difference between any two populations of average points from FLIM phasor distributions. Scatter plot of 17 average points from the FLIM phasor distributions corresponding to the varying levels of melanin pigmentation within fixed HCMs (n = 3) segmented by 7 different colored phasor clusters. The SD for each population of average points was generated. Smaller error bars were determined for average points associated with FLIM phasor clusters of higher FLIM intensity. Larger error bars were observed for average points associated with FLIM phasor clusters of lower FLIM intensity. The difference, in terms of s values, between any two populations of average points was statistically significant (t test, P < 0.05). SD = Standard deviation, G = X coordinate of phasor transform (‘real’ unitless phasor component), S = Y coordinate of phasor transform (‘imaginary’ unitless phasor component), Scale bar = 10 μm.

The average points and standard deviation from the FLIM phasor distributions corresponding to the heterogenous melanin pigmentation within fixed HCMs (n = 3) segmented by 7 different colored phasor clusters were generated and plotted. The difference, in terms of s values, between any two populations of average points was statistically significant (t test, P < 0.05) (Fig. 5). The fluorescence decay measured from the eumelanin-enriched dark brown hair, dark pigmented HCM, the pheomelanin-enriched red hair and light pigmented HCM were fitted using a two exponentials model for comparison (Supplementary Table 5). We observed that the average lifetimes (intensity) measured from the dark brown hair (1.7 ± 0.012 ns) was comparable with the dark pigmented HCM cell (1.3 ± 0.012 ns) while the average lifetimes (intensity) measured from the red hair (2.4 ± 0.0091 ns) was comparable with the light pigmented HCM cell (2.1 ± 0.011 ns). Taken together, the spectral phasor analysis from the HCM intracellular melanins had generally poorer signal to noise ratio than the FLIM phasor and as such, was less able to separate the two melanin species due to spectral overlap. Descanned detection has a poorer signal to noise ratio even when data were acquired from the same field of view and acquisition parameters (Supplementary Figure 5). In addition, the segmentation of the spectral phasor distribution from the darker eumelanin-enriched cytoplasm was more difficult than the lighter pheomelanin-enriched cytoplasm because of the poorer signal to noise data from the darker cells.

### FLIM phasors derived from HCM melanins are distinct from measured cytoplasmic NADH

The prominent endogenous fluorophore within HCMs is melanin (eumelanin/pheomelanin), the dark colored eumelanin characterized by a short fluorescence lifetime and the light pigmented pheomelanin by a long fluorescence lifetime^25^. The 2PM FLIM and resultant phasors acquired from heterogeneously pigmented HCMs were expected to be linearly distributed for phasors mapped between eumelanin and pheomelanin-dense melanin mixtures. However, HCMs also harbor the ubiquitous endogenous fluorophore, cytoplasmic NADH, found within the mitochondrial inner membranes^34^. NADH is acknowledged as one of the strongest natural intracellular fluorophores and optimally 2-photon excited at 740 nm^25,35^. We further investigated the potential impact of cytoplasmic NADH on the linear distribution of the intracellular melanin-derived FLIM phasors by comparing phasor distributions for fixed (pigmented) HCMs and fixed (non-pigmented) HEK293 cells.

**Figure 6:**
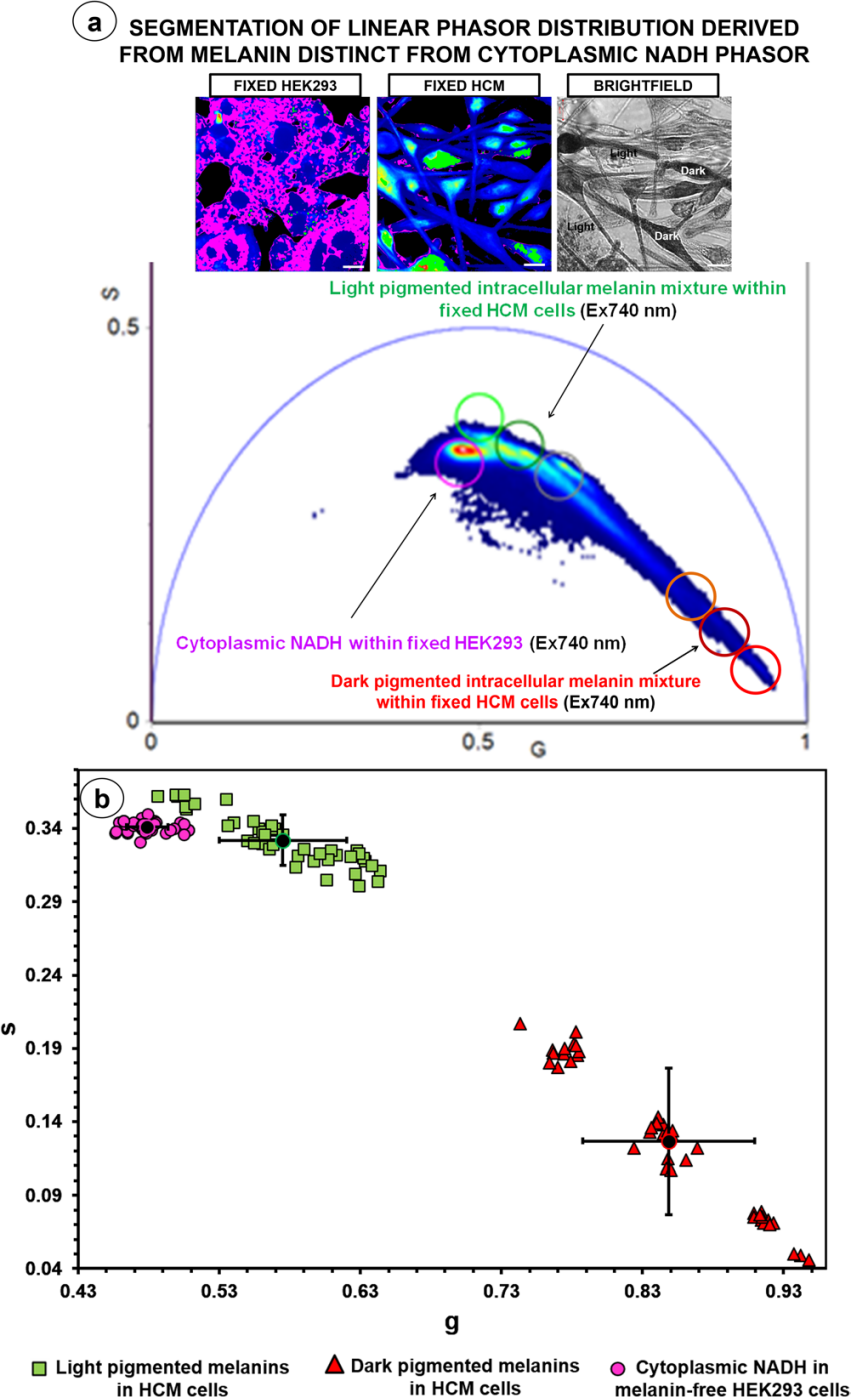
2PM FLIM phasor distribution from heterogeneous pigmented intracellular melanin was statistically distinct but proximal from cytoplasmic NADH phasor distribution. Cytoplasmic NADH within the melanin-free HEK293 cells was optimally excited at 740 nm, and 2PM FLIM data acquisition and phasor analysis were also performed at this wavelength on fixed HCMs. (**a**) The merged FLIM phasor distribution showed that the phasor distribution identified from cytoplasmic NADH and light pigmented melanins in HCMs (n = 3) was distinct. (**b**) Scatter plot of 42 average points from the FLIM phasor distributions corresponding to the light and dark pigmented melanins within fixed HCMs (n = 3), and cytoplasmic NADH. The SD for each population of average points was generated. The difference, in terms of s values, between the light pigmented melanin and cytoplasmic NADH populations was statistically significant (t test, P = 0.002). The difference (in s values) between the dark pigmented melanin and cytoplasmic NADH populations was also statistically significant (t test, P < 0.05). SD = Standard deviation, G = X coordinate of phasor transform (‘real’ unitless phasor component), S = Y coordinate of phasor transform (‘imaginary’ unitless phasor component), Scale bar = 10 μm.

When the phasor plots from fixed HCM and HEK293 cells were examined at 740 nm, the segmented phasor clusters mapping to the light pigmented melanin mixture and cytoplasmic NADH were distinct and positioned close to each other (Supplementary Figure 6a). The phasor distribution of the intracellular melanins from HCMs was linear, however the adjacent phasor clusters mapping to both the light pigmented pheomelanin-enriched HCM content and cytoplasmic NADH may lead to non-linearity of the phasor pixel distribution (Fig. 6a). The difference, in terms of s values, between the two populations of average points corresponding to the light pigmented melanins within fixed HCMs (n = 3) and cytoplasmic NADH within fixed HEK293 cells was statistically significant (t test, P = 0.002) (Fig. 6b). For completeness, the difference (in s values) between the two populations of average points corresponding to the dark pigmented melanins within fixed HCMs (n = 3) and cytoplasmic NADH was also statistically significant (t test, P < 0.05) (Fig. 6b). Similar observations were made for fixed HCM and HEK293 cells examined at 780 nm, optimal for the intracellular melanin excitation (Supplementary Figure 6b). The linear phasor distribution acquired from HCMs at 740 nm (optimal NADH excitation wavelength) and 780 nm (optimal melanin excitation wavelength) overlapped, confirming that the linear phasor distribution was maintained at both 740 nm and 780 nm. The FLIM phasor distributions of unfixed and fixed HEK293 cells also overlapped providing evidence that cytoplasmic NADH could be successfully measured in paraformaldehyde-fixed cells (Supplementary Figure 6c).

### FLIM phasor clusters mapping to Melan-A Ab3/Alexa Fluor 594 fluorophore are confined to lightly pigmented HCMs

**Figure 7:**
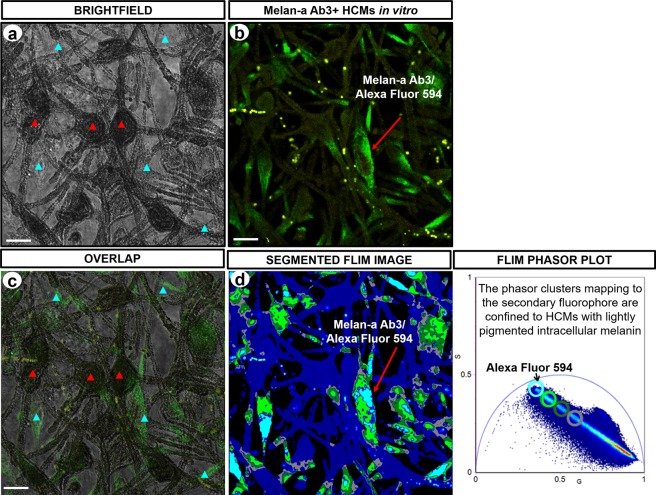
Imaging of immunolabeled cultured HCMs. (**a**) Brightfield image of fixed HCMs *in vitro*. Red arrow head indicates cellular regions with very dark pigmented melanin. Blue arrow head indicates cellular regions with lightly pigmented melanin. (**b**) Image of fluorescence emission from HCMs showing melanosome positive Melan-A Ab3/Alexa Fluor 594 immunolabeling. Figure (**c**) displays the overlapped images of (**a**,**b**) that identifies the cellular regions where fluorescence emission can be detected. Fluorescence emission was clearly detected in light pigmented cellular regions, however, emitted fluorescence was limited in dark pigmented cellular regions, identified with a high eumelanin/pheomelanin ratio. (**d**) Image of segmented FLIM phasors derived from intracellular melanin of the immunolabeled HCMs. Phasor clusters mapping to the Melan-A Ab3/Alexa Fluor 594 immunolabel were confined to HCMs with lightly pigmented melanins. G = X coordinate of phasor transform (‘real’ unitless phasor component), S = Y coordinate of phasor transform (‘imaginary’ unitless phasor component), Scale bar = 10 μm.

HCMs were immunolabeled with melanosome positive Melan-A Ab3/Alexa Fluor 594 and processed under the same conditions. As the melanin-containing melanosomes are distributed in the entire cellular cytoplasm, we expected to consistently detect immunofluorescence from all regions of HCMs upon excitation. However, when the image of the immunolabeled HCMs (Fig. 7b) and the brightfield image (Fig. 7a) were merged, we detected limited immunofluorescence intensity from dark pigmented regions of the HCMs compared to the light pigmented HCMs (Fig. 7c). This suggests that the detection of immunofluorescence is limited or obstructed by the dark melanins. The segmented phasor distribution of the same field of immunolabeled HCMs (Fig. 7d) showed the heterogeneously pigmented HCMs to be consistently identified by the melanin content. This confirmed that phasor clusters mapping to the Melan-A Ab3/Alexa Fluor 594 fluorophore were sequestered to cytoplasmic regions of HCMs with lightly pigmented melanins (Fig. 7b).

### Profiling of intracellular HCM melanins and surrounding choroidal components

**Figure 8:**
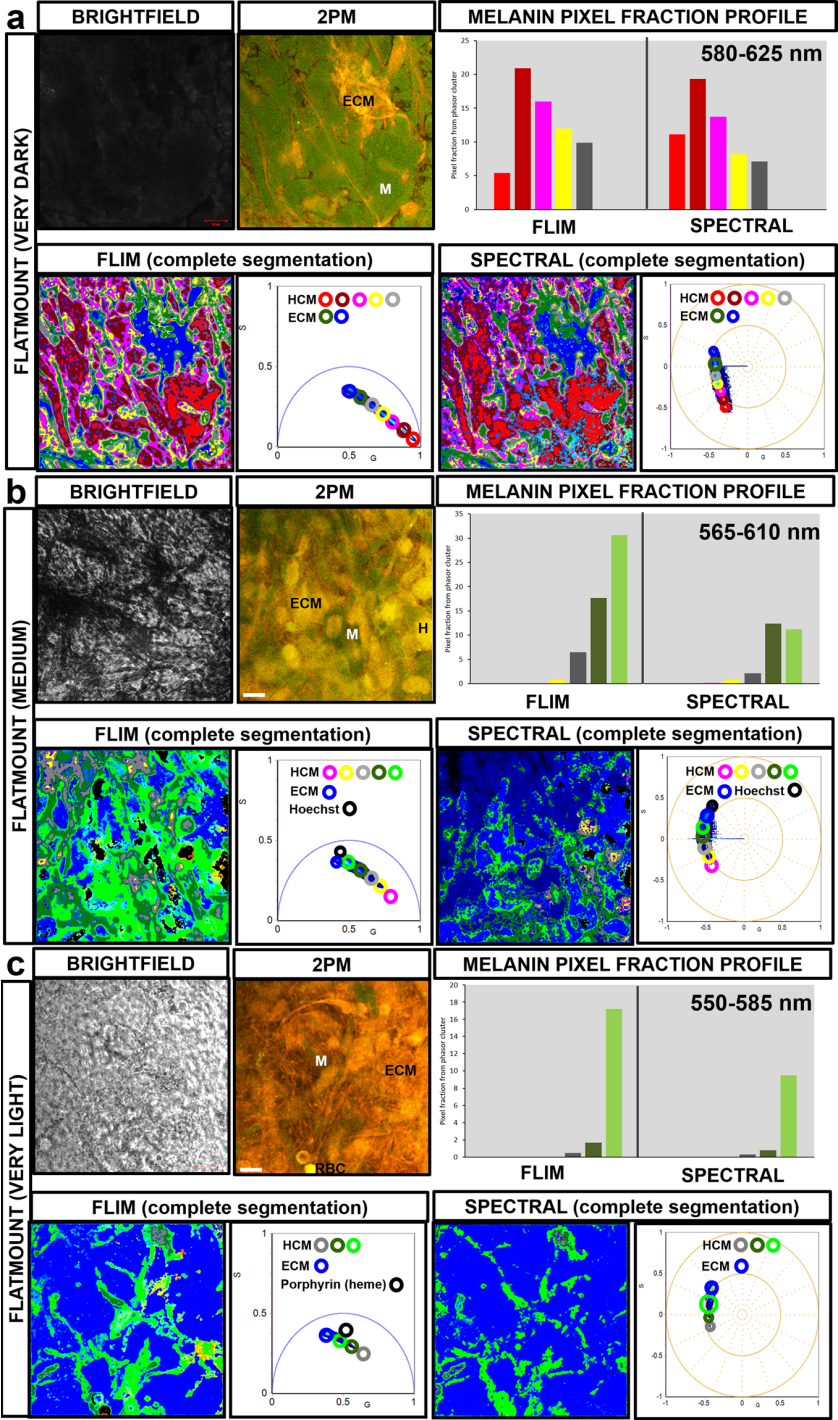
2PM FLIM/spectral phasor distribution mapping to HCM-localized melanins and surrounding human choroidal flatmount tissue. The image segmented FLIM/spectral phasor clusters mapping to (**a**) ‘very dark’ pigmented HCMs displayed short average fluorescence lifetimes/emission spectra: 580 to 625 nm. FLIM/spectral phasor clusters mapping to (**b**) ‘medium’ pigmented HCMs had longer fluorescence lifetimes/emission spectra: 565 to 610 nm. FLIM/spectral phasor clusters mapping to (**c**) ‘very light’ HCMs showed the longest fluorescence lifetimes/emission spectra: 550 to 585 nm. The unmixed FLIM phasor clusters mapped to fluorophores contained in ECM, RBCs and Hoechst 33342 nucleic DNA binding stain were distinct from the HCM-matched clusters but image segmented spectral phasor clusters showed overlapping emission spectra. M = HCM, ECM = Extracellular matrix, H = Hoechst 33342, RBC = Red blood cell, G = X coordinate of phasor transform (‘real’ unitless phasor component), S = Y coordinate of phasor transform (‘imaginary’ unitless phasor component), Scale bar = 10 μm.

Intracellular melanins in label-free and fixed HCMs in choroidal flatmounts (Fig. 8) and cross-sections (Supplementary Figure 8b) were examined using FLIM/spectral phasor segmentation and intracellular melanin profiling. The quantitative profile of intracellular melanins in HCMs cells based on spectral and FLIM phasors is provided in Supplementary Figure 8a. When the FLIM and spectral image of a ‘very dark’ pigmented flatmount was segmented by mapping phasor clusters to the HCM-localized melanins (Fig. 8a), the phasor clusters that dominantly mapped to the intracellular melanins were clusters with shorter average fluorescence lifetimes (**cluster i-iv**) and emitted fluorescence between 580 to 625 nm. When FLIM and spectral images of ‘medium’ pigmented flatmount (Fig. 8b) and cross-section (Supplementary Figure 8b) samples were segmented, the prominent phasor clusters had longer average fluorescence lifetimes (**cluster iii-vii**) and emitted fluorescence between 540 to 610 nm. When the FLIM and spectral image of a ‘very light’ pigmented flatmount (Fig. 8c) was segmented, **cluster vii** was the dominant phasor cluster identified, with the longest average fluorescence lifetime and emitted fluorescence between 550 to 585 nm. FLIM phasor clusters also mapped to surrounding choroidal fluorophores such as ECM (collagens and elastins), RBCs (heme) and Hoechst 33342 nucleic DNA binding stain (Fig. 8a,b,c). These were clearly distinct from HCM intracellular melanin phasor clusters. Segmented spectral phasor clusters mapping to HCM intracellular melanins also mapped to other choroidal structures such as ECM due to the overlapping fluorescence emission spectra. When the fraction percentage of image pixels representing intracellular melanin per total sample field image pixels was investigated, based on image segmented FLIM and spectral phasor plot, the FLIM data (non-descanned mode) gave a higher signal to noise ratio than the spectral emission data (Supplementary Figure 8c).

## Discussion

In this study, we visualized and quantified spatial profiles of cytoplasmic melanins (eumelanin/pheomelanin) and melanosomes within label-free *in vitro and in situ* HCMs. We achieved this by combining 2PM fluorescence spectral emission and lifetime imaging with phasor plot segmentation, avoiding tissue-destructive melanin extraction processes such as absorption spectrum analysis^36^ and HPLC^17,18^. This non-invasive approach consistently confirmed intracellular melanin as an endogenous fluorophore that is a reliable biomarker for variably pigmented HCMs.

Although label-free dark-colored HCMs can be easily recognized in tissue by their pigmentation and general morphology, visualizing all choroidal melanocytes *in situ* can be limited related to their heterogeneous pigmentation, cellular morphology and complex extracellular microenvironment^1^. Red chromogen-based immunohistochemistry is usually performed on melanocytic/melanoma tissue with abundant brown pigmentation for diagnostic pathology^37^. An alternative approach using label-free imaging of melanin autofluorescence could potentially extend our knowledge of melanin species (eumelanin/pheomelanin) composition and provide spatial information of melanocytes and cytoplasmic melanosomes.

FLIM and spectral phasor segmentation provided an alternative novel approach for quantitative and spatial studies of cytoplasmic eumelanins and pheomelanins within label-free and fixed *in vitro* and *in situ* HCMs^25,28^. We performed spectral and FLIM imaging at 780 nm, the optimal excitation wavelength for melanins, consistent with the 2PM excitation wavelengths applied previously for melanins (between 720 to 880 nm)^38^. Other complementary non-destructive and non-invasive approaches have been used to study the intracellular melanin content in skin and hair (Supplementary Note 9). The limitations of these techniques added gravitas to our decision to apply 2PM FLIM and spectral phasor plot techniques in this study.

The first step in applying these techniques to choroidal melanocytes (and intracellular melanins) was to profile the emission spectra and lifetimes of controls for melanins and choroid fluorophores. The controls for image phasor segmentation encompassed varying levels of melanin: eumelanin-enriched (dark pigmented synthetic melanin, human hair and choroidal flatmount), mixed eumelanin/pheomelanin (red-brown bird feather) and pheomelanin-enriched (light pigmented human hair, amelanotic melanoma and fetal choroid flatmount). Although these controls do not completely replicate the choroid microenvironment, they provide a frame of reference for intracellular melanin lifetimes and spectra. Synthetic melanin and human hair have been previously used as melanin controls^23,25,39^. Supplementary Note 10 reviews the use of a red-brown feather, amelanotic human eye melanomas and fetal choroid for melanin controls. The dark pigmented melanin controls were associated with short average fluorescence lifetime phasor clusters and red-shifted longer emission spectra, while lighter pigmented melanin controls mapped to longer average fluorescence lifetime phasor clusters with shorter emission spectra. The FLIM-phasor clusters mapping to ‘very dark’ to ‘very light’ melanin controls were linearly distributed and distinct from the ECM and heme fluorophores, although the emission spectra did appear to overlap.

With profiling of controls established, 2PM spectral and FLIM phasor profiling of intracellular melanins in label-free and fixed HCM cells was performed. The image segmentation of the HCM-derived spectral phasor distribution was carefully matched with the intracellular melanin spatial mapping in the FLIM phasor segmentation and this yielded several significant observations. First, the intracellular melanin-mapped FLIM phasors showed a linear distribution confirming the intracellular melanins were essentially comprised of two fluorophore species^40^, presumably eumelanin and pheomelanin^41^. The linear combination of the phasor allows for the decomposition of individual species that have complex decay kinetics. The relative contribution of those components can be obtained graphically by calculating the distance between the combination point in the phasor plot and the individual component positions in the universal circle where the ratio of the linear combination determines the fraction of the components. This is one of the major advantages of using phasor analysis on complex samples such as melanin. Phasor analysis requires no pre-requisite knowledge of the original decay allowing conclusions based on the linearity of the distribution. This technique has been demonstrated in differentiating bound and free NADH^35,42^. In addition, the examined individual species do not need to be a single-exponential species. This is a key concept in applying the phasor plot to analyze a complex system, where it is almost impossible to solve individual lifetime components^43^.

In our HCM samples, the melanins were previously shown by Wakamatsu *et al*. (2008) to be a mixture of eumelanin and pheomelanin using destructive chemical degradation and microanalytical HPLC^17^. Phasor analysis has been previously used by Krasieva *et al*. (2013) to study the relative contributions of eumelanin and pheomelanin in skin melanocytes^25^. They showed that each point along the linear cluster combination has a color that mapped to a specific relative concentration of the melanin species and also showed that eumelanin enriched dark hair has a short fluorescence lifetime and pheomelanin enriched red hair has a longer fluorescence lifetime using FLIM fingerprinting of dark and red human hair^25^.

Second, the image segmented FLIM phasor clusters with short average lifetimes mapped to dark pigmented regions of HCMs and eumelanin-enriched controls, confirming the cellular regions were eumelanin-dense. FLIM phasor clusters with longer average lifetimes were associated with lighter pigmented cellular regions and higher pheomelanin content controls, verifying much reduced eumelanin/pheomelanin ratio previously reported in these regions^25,44^. Mapping of short and long average lifetime clusters to dark eumelanosomes and light pheomelanosomes was previously described for melanins and melanosomes within skin and hair melanocytes^25,44^. The dark pigmented regions of HCMs and eumelanin-enriched controls mapped to red-shifted longer spectral emission phasor clusters, while the lighter pigmented HCM regions and melanin controls with a lower eumelanin/pheomelanin ratio mapped to shorter spectral emission phasor clusters. The shift in peak emission identified between dark eumelanin-enriched and light pheomelanin-enriched melanocytes of around 45 nm is greater than a melanin concentration dependent shift of around 10 nm.

Third, based on the merged FLIM phasor profiling of fixed and melanin-free HEK293 cells, the FLIM phasor clusters mapping to the ubiquitous cytoplasmic NADH and the lightly pigmented melanin showed statistically distinct (but proximal) positions in the phasor plot.

Fourth, we observed co-localization of melanosomes with dark melanins (shorter average lifetime clusters) and those with light melanins (longer average lifetime clusters), in HCM perinuclear locations and peripheral regions of cell processes. Based on studies of melanosomes within mammalian skin melanocytes^45–48^, the cytoplasmic melanosome distribution is thought to depend on complex organelle-vesicle transportation highways comprised of long-acting bidirectional microtubules, short-acting unidirectional actins and motor proteins that include kinesins, dyneins and myosins^10,46–48^.

Our results also showed a higher signal to noise ratio for the acquired 2PM FLIM data from cells and tissues as compared to the mode of acquisition for emission spectra (Supplementary Note 5). This could be explained by the process of photon acquisition for these approaches, and the biophysical characteristics of melanins. The emitted light in the FLIM non-descanned detection is collected directly from the sampled specimen through an external detector with reduced path length, less optical elements (mirrors and lenses) and more scattered photons collected^49^. In comparison, spectral descanned detection incurs substantial light loss as the emitted light returns along the same path as the excitation light, hitting the scanning mirrors before reaching the detector within the scan head^49^. Additionally, scattered emission photons in spectral detection cannot be collimated and thus not focused back through the beam path of the scan head. This has previously been acknowledged as an issue with spectral detection in tissue imaging^50^.

With regards to the biophysical characteristics of melanin species, the ionization potential of eumelanin is higher than pheomelanin, requiring greater laser power to increase the number of photons detected per image pixel, despite using the melanin-optimal 780 nm excitation wavelength^51–54^. We observed, however, that dark pigmented HCMs are most susceptible to photodamage even at low laser power, limiting the acquisition of photons for spectral imaging^54^. As such, we applied a low laser power (under 5%) to keep photodamage of our fixed biological specimens (HCM cells and choroid tissues) to a minimum. The same effect has been observed when examining the fluorescence emission spectra from skin and hair samples with high eumelanin content and was suggested to be due to eumelanin one-photon absorption of near infrared light^25^. The fluorescence emission yield from eumelanin could also be affected by eumelanin aggregation observed within HCMs; a previous study reported that the fluorescence emission yield detected from large aggregates of eumelanin molecules was 5.7 times less than for small eumelanin aggregates^55^. This makes 2PM FLIM a more effective approach to analyze dark pigmented HCMs as FLIM acquisition is largely independent of the fluorophore concentration, absorption of the sample, sample thickness, photo-bleaching and/or excitation intensity^56,57^.

Taken together, these factors can explain why the quality of 2PM FLIM phasor segmentation from the specimens (cells, tissue flatmounts and cross-sections) was generally better in the current study than spectral phasor segmentation. The higher number of photon counts acquired in each pixel from 2PM FLIM results in a tighter distribution of phasor points with similar lifetimes. The variance of the phasor positions is entirely dependent on the inverse square root of the number of photons recorded^58^. As such, it provides a robust characterization of HCMs and choroid endogenous fluorophores (including melanins).

The image pixel fraction associated with the image segmented FLIM and spectral phasor clusters could also be used to generate quantitative histogram profiles of HCM intracellular melanins. These profiles revealed the prominent melanin species within each sample region and allowed, for the first time, an approach to quantify eumelanin to pheomelanin ratios in HCM cells and choroid tissues without tissue destruction. Our study also provided insights for melanosomes within eye melanocytes; this area is neglected in eye melanocyte research. This melanin profiling strategy is a significant outcome and can be directly translated to *in situ* studies of other melanocytic lesions in the eye and other tissues.

The application of 2PM strategies also provided consistent visualization of HCMs with endogenous dark and light melanin fluorophores, regardless of the density of tissue pigmentation. Immunolabeled HCMs with abundant levels of dark pigmented melanins may not always be consistently detected despite using effective fluorescent IHC protocols. Substantial intracellular melanin levels have been previously implicated in interfering with fluorescence emission from targeted cellular antigens^59^. Melanins within the melanosomes can absorb fluorescence emission from excited fluorophores^59^ and have been shown to physically obstruct the cellular and tissue morphology of heavily pigmented melanocytic tissues^60^. As such, the application of 2PM benefits HCM identification without relying solely on melanocyte-specific immunolabeling visualized with fluorescence or red chromogen-based immunohistochemistry^13,23,37^. Furthermore, 2PM imaging of HCMs visualizes cytoplasmic melanosomes, an important distinguishing feature for differentiating between HCMs and melanin-filled choroidal macrophages *in situ*, as macrophages do not have melanosome organelles^61,62^.

In conclusion, the application of 2PM FLIM/spectral phasor analyses to human eye choroid provides a comprehensive and robust ‘model-free’ examination of biochemical and spatial variations of melanins and melanosomes in heterogeneously pigmented melanocytes (*in vitro* and *in situ*). Combined multidimensional spectral and FLIM phasor approaches may, in some circumstances, provide improved segmentation of fluorophores^27^, however based on the current results, FLIM phasor analyses more clearly segmented HCM intracellular melanin mixtures *in situ*. This included consistent quantitative profiles for cytoplasmic melanin species (eumelanin/pheomelanin) and cellular spatial distribution of melanosomes. An apparent shortcoming of the phasor approach is the lack of absolute decay times and amplitudes with molecular species and their relative abundances. In such a scenario, computationally complex exponential fitting or spectral unmixing would be required^63^. The new observations in this study further our understanding in establishing optimal imaging parameters for identifying and characterizing melanins. FLIM and spectral phasor analysis could also be used to better characterize the unique cellular and extracellular microenvironment of eye melanocytic lesions.

## Methods

### Sample preparation

#### Human choroidal tissue

*Post-mortem* human eyes were obtained from the Lions NSW Eye Bank, with informed consent from the eye donor next of kin, and approval from the University of NSW Human Research Ethics Committee (HREC #HC14261). All methods applied in the study were performed in accordance with the approved Human Research Ethics guidelines.

Eyes without corneas were fixed in 2% paraformaldehyde (Sigma-Aldrich, Australia) in phosphate buffered saline (PBS) (pH 7.4) within 18 hours *post-mortem*. A circumferential incision was made around the edge of the ciliary body, and the iris, lens, ciliary body and vitreous were removed. The fixed eye with intact retina, choroid and sclera was used to prepare either choroidal flatmounts (n = 8) or paraffin embedded tissue-cross sections (n = 3). All processed samples were mounted in 100% glycerol.

#### Cell culture and chamber slide seeding

Primary HCMs were cultured from *post-mortem* human choroidal tissue (n = 3, aged 59–77 years) as previously described^64,65^ with minor modifications. HCMs were isolated and cultured in melanocyte growth medium [10% fetal bovine serum (FBS) in Ham’s F12 with 2mM L-glutamine, 0.1 mM Isobutylmethylxanthine (IBMX) (Sigma-Aldrich), 100 nM Phorbol 12-myristate 13-acetate (PMA) (Sigma-Aldrich), 10 ng/ml cholera toxin (Sigma-Aldrich)], seeded into 8-well chamber slides (Merck Millipore) and cultured for at least three days, rinsed twice in PBS and fixed with 2% paraformaldehyde in PBS (pH 7.4) at room temperature for 30 minutes.

#### Choroidal flatmounts

Retina-choroid tissue punches from fixed eyes (n = 8, aged 49–76 years) were taken (without puncturing the outer sclera) using a 2 mm biopsy punch (Kai Medical) in central choroid (sub-macula, near the optic disc). Using a dissecting microscope (Leica M60), tissue punches of retina-choroid were carefully separated from the underlying sclera using curved forceps and paintbrush, and the retina carefully separated from the adjacent RPE-choroid in PBS. The flatmounts were then carefully oriented suprachoroid upwards on Super-Frost Plus (Menzel-Glaser) slides, cover slipped with 80% glycerol/PBS and sealed with nail polish.

#### Paraffin cross-sections

Fixed human eyes (retina-choroid-sclera) (n = 3, age: 59–72 years) were processed and paraffin wax-embedded, and cross-sections cut at 7 µm thickness. Sections were collected on Super-Frost Plus (Menzel-Glaser) slides. Paraffin sections were dewaxed through xylene and alcohols to water, then air dried.

A summary of the donor sex, age, cause of death, ocular and systemic conditions is provided in Table 1.

**Table 1:** Demographic information, cause of death, medical record of human eye donors.

#	Sample preparation	Sex	Age (years)	Cause of death	Ocular conditions	Systemic conditions
1	Cell culture	Male	78	Spontaneous Intracerebral Haemorrhage (surgery), Hypertension	Not known	Aortic mitral regurgitation, Aortic stenosis, Aspirin, Bowel cancer, High Cholesterol, Bilateral pleural effusion, Transitional cell carcinoma bladder cancer, Benign prostatic hyperplasia, Non-malignant lung lesion
2	Cell culture	Male	77	Congestive heart failure	Not known	Lower respiratory tract infection, Ischaemic heart disease, Atrial Fibrillation
3	Cell culture	Female	51	Metastatic endometrial cancer	Not known	Peritoneal metastasis, Hemithyroidectomy, High cholesterol
4	Cross section	Male	72	Cardiac arrest	Not known	Heart disease, Cardiac vascular
5	Cross section	Male	66	Thalamus, Internal Capsule Haemorrhage with Intraventricular Extension, Midline shift	Wore glasses	Large intracerebral haemorrhage, Gout
6	Cross section	Female	59	Metastatic lung cancer	Not known	Metastatic lung cancer
7	Flatmount	Female	76	Colorectal cancer	Not known	Not known
8	Flatmount	Female	74	Metastatic non-small cell lung cancer	Not known	Metastatic non-small cell lung cancer, Squamous cell carcinoma, Pulmonary embolism
9	Flatmount	Female	69	Hypoxic brain injury, Cardiac arrest, Respiratory failure	Not known	Not known
10	Flatmount	Male	62	Lung adenocarcinoma	Not known	Type 2 diabetes mellitus, Metastatic lung mass (non-small cell lung cancer) from colonic cancer, Hyptertension, Asthma
11	Flatmount	Male	59	Anaphylaxis	Not known	Cerebral hypoxia secondary to anaphylaxis, Ear cancer
12	Flatmount	Male	57	Hepatic failure secondary to metastatic breast cancer	Not known	Not known
13	Flatmount	Male	53	Cardiac arrest	Not known	Chest pain, Mini stroke, Hypercholesterolemia, Hypertension, Diabetic
14	Flatmount	Female	49	Metastatic melanoma	Not known	Lung cancer, liver cancer, bone cancer

The delay to fixation for the human eye tissue was within 18 hours post-mortem.

### 2PM excitation spectral emission and FLIM imaging

2PM requires the simultaneous absorption of two photons in a single quantitized event^66^. 2PM spectral emission and FLIM imaging was acquired on a Zeiss LSM 880 microscope using a Mai Tai Insight DeepSea 2Ptunable laser operating at 80 MHz repetition rate and a Plan-Apochromat 63.0 × 1.4 Oil objective (Zeiss-GmbH, Germany). TimeHarp 260 board was utilized to acquire fluorescence decay data using Time-Correlated Single Photon Counting (TCSPC). For single plane flatmount imaging of HCMs, the microscope objective was focused to the posterior suprachoroid plane of the eye choroid tissue. As the choroid layer varies in thickness between tissue flatmounts, we acquired the most focused plane of HCMs in the suprachoroid which was oriented upwards and placed closest to the cover slip. The suprachoroid is the outermost layer of the choroid and a transition zone between the choroid and outer sclera. It mostly contains collagen fibers and melanocytes interspersed between fibroblasts^1^.

Lambda (ʎ) spectral imaging mode was configured in 32 channels (32 channel GaAsP spectral detector), each with 8.9 nm spectral bin width and a total range between 410 nm and 696 nm. All spectral data were collected over the whole spectrum in increments. This allowed the unmixing of spectral signals even though they are overlapping. FLIM measured from HCMs and choroidal tissue was detected by GaAsP non-descanned detectors (Zeiss BiG.2 type A module with two GaAsP detectors; BP 500–550 nm; BP 575–610 nm). Both spectral emission and FLIM imaging were acquired with 1024 × 1024-pixel frame. The pixel dwell time for FLIM imaging was 8.19 µs/pixel and 32.8 µs/pixel for spectral imaging. The calibration of the lifetime measurements was performed using 1 μM ATTO 488/milli-Q water solution (ATTO-TEC GmbH, Siegen, Germany). The spectral emission and FLIM imaging data were analyzed using a FLIM phasor plot approach using the SimFCS software, developed at the Laboratory of Fluorescence Dynamics (UC Irvine).

### Spectral phasor plot segmentation

Spectral phasor approach provides an advantage that the entire spectrum is not needed for some of the classical spectral analysis techniques such as species demixing. Only a few parameters of the spectral distribution are sufficient for these calculations^67^.

The fluorescence spectra at every pixel of the spectral image were transformed into two coordinates in a Cartesian spectral phasor plot as described by the following equations^27^.

$${\rm{X}}-{\rm{coordinate}}=g({\rm{\lambda }})=\frac{{\int }_{\lambda min}^{\lambda max}I(\lambda )\cos (\frac{2\pi n(\lambda -\lambda {\rm{i}})}{\lambda max-\lambda min})d\lambda }{{\int }_{\lambda min}^{\lambda max}I(\lambda )d\lambda }$$

$${\rm{Y}}-{\rm{coordinate}}=s({\rm{\lambda }})=\frac{{\int }_{\lambda min}^{\lambda max}I(\lambda )\sin (\frac{2\pi n(\lambda -\lambda {\rm{i}})}{\lambda max-\lambda min})d\lambda }{{\int }_{\lambda min}^{\lambda max}I(\lambda )d\lambda }$$

I(λ) represented the intensity at every spectral wavelength (channel), n was the harmonic number and λ_i_ was the initial wavelength. The phasors were plotted in the four-quadrant spectral phasor plot with the origin in the (0,0) point. The phasor for the background signal which can be considered as a spectrum with infinite spectral width was located at the center of phasor with coordinate (0,0)^68^. The position of every pixel in the spectral phasor plot was described by phase angle (*φ* and modulus (M).

$$\varphi =\arctan (\frac{s({\rm{\lambda }})}{g({\rm{\lambda }})})$$

$$M=\sqrt{{{s}^{2}}_{({\rm{\lambda }})}+{{g}^{2}}_{({\rm{\lambda }})}}$$

The angular position in the spectral phasor plot relates to the center of mass of the emission spectrum and the modulus depend on the spectrum’s full width at the half maximum^27^. If the spectrum is broad, its location should head towards the center of the plot. If there is a red shift in the emission spectrum, its location will move counter clockwise toward increasing angle from position (1,0). Spectral phasors follow rules of vector addition and orthogonality like the lifetime phasors. The method only requires the zero and first order of the spectrum, which allows for the rapid and reliable unmixing of spectrally overlapping fluorophores within biological samples. Therefore, the analysis is not affected by spectral bleed and as such, is suitable for resolving overlapping spectra^68^. It also doesn’t require spectral separation as long as the shape of the spectra are different; they can be unmixed even with peak overlap.

Colored-phasor selection clusters were used to segment the spectral phasor distribution^27^. The fluorescence spectra corresponding to the relative mixtures of eumelanin and pheomelanin within the sampled HCMs were analyzed on a pixel by pixel basis. After the spectral phasor distribution has been segmented by mapping phasor clusters to regions of interest in the image, the analyzed phasor plot is referred as segmented phasor plot. The peak emission wavelength of the selected cursor’s center of mass were determined. All pixels selected by the cursor have similar peak wavelengths.

The image segmentation of the cytoplasmic melanin spectral phasors was spatially matched with the image segmentation of the FLIM phasors by mapping the segmented spectral phasor clusters to the same HCM regions segmented by FLIM phasor clusters. This enabled us to compare the quality of the phasor segmentation results from both techniques.

### FLIM phasor plot segmentation

In brief, the fluorescence decay curve at every pixel of the FLIM image was transformed into two coordinates in a Cartesian lifetime phasor plot using the rules of Fourier phasor transformation^69^. The coordinates were calculated based on the following equations:

$${\rm{X}}-{\rm{coordinate}}=g(\omega )=\frac{{\int }_{0}^{\infty }I(t)\cos (\omega t)dt}{{\int }_{0}^{\infty }I(t)dt}$$

$${\rm{Y}}-{\rm{coordinate}}=s(\omega )=\frac{{\int }_{0}^{\infty }I(t)\sin ({\rm{\omega }}t)dt}{{\int }_{0}^{\infty }I(t)dt}$$

where *g*(*ω*) and *s*(*ω*) were the X and Y coordinates of the phasor transforms. *ω* was the angular repetition frequency (*2πf*) of the excitation source where *f* was the laser repetition rate^28^. Phasors were plotted as a two-dimensional phasor plot with the origin in the (0,0) point. The horizontal axis represented the ‘real’ (g) phasor component with values between 0 and 1. The vertical axis represented the ‘imaginary’ (s) phasor component with values from 0 and 0.5. The coordinates have no units as phasors were normalized^28,40^.

All single fluorophore-derived phasors must lie on the universal circle of the phasor plot. A two-component phasor must be located on a line between the fluorescence lifetimes of the two individual fluorophore species (*τ*1 and (*τ*2) with its position calculated by the weighting of the two lifetimes. Multi-component phasors contributed by the linear combination of multiple single component phasors are distributed inside the universal circle^42^ where phasors follow rules of vector addition and orthogonality^27^. If the phasor is not on the universal circle, the corresponding molecular species must have a complex multi-exponential decay^42^. In general, the overall decay in a sample with multiple fluorescent species is a phasor that is the sum of the independent phasors of each fluorescent species^42^.

The linear combination of the phasors does allow for the decomposition of individual species that have complex decay kinetics. The relative contribution of the components can be obtained graphically by calculating the distance between the combination point in the phasor plot and the individual component positions in the universal circle i.e. the ratio of the linear combination determines the fraction of the components. For example, two molecular species with multi-exponential decay are identified by two specific points in the phasor plot inside the universal circle. All possible weighting of the two molecular species give phasors distributed along a straight line joining the phasors of the two species^42^. For three or more molecular species, all of the possible combinations contained in a triangular or polygonal plot can still establish a straight line if the vector summation of the multiple fluorescent species gives phasors that are linearly distributed. The vertices of the line correspond to the specific phasor of the contributing species^42^. This is one of the major advantages of using phasor analysis on complex samples such as melanin biomolecules. This property allowed for a ‘model-free’ approach in identifying areas in the lifetime image with similar or different lifetimes^70^. It allows us to make conclusions based on the linearity of the distribution. In addition, the examined individual species do not need to be a mono or single-exponential species to display a linear phasor distribution. This is a key concept in applying the phasor plot to analyze a complex system, where it is almost impossible to solve individual lifetime components^43^.

Transformed fluorescence lifetimes distributed close to the (1,0) point were short and longer lifetimes were distributed away from this point within the universal circle. The E-filter (median convolution filter) procedure of SimFCS was applied to the resultant phasor plots so that the variance of the phasor position could be reduced without reducing the image resolution. Phasors measured at the image border were not filtered in this convolution procedure^28^.

Colored phasor selection clusters were used to segment the derived lifetime phasors by mapping them to specific image pixels of the sampled tissue such as the HCM intracellular melanin content. Mapped image pixels were color-tagged with the color of the applied selection clusters. Multiple cursors were used to select multiple phasor clusters within the phasor plot, allowing the spatial locations of these selections to be visualized. The fluorescence lifetimes corresponding to the relative mixtures of eumelanin and pheomelanin within the sampled HCMs were analyzed on a pixel by pixel basis. The size and positioning of the cursors depended on the specific phasor distribution being analyzed.

To reduce selection bias, the phasor plot segmentation process referred to a well-focused FLIM image of the tissue morphology and FLIM phasor profiling of melanin-choroidal fluorophore controls. After the FLIM phasor distribution has been segmented by mapping phasor clusters to regions of interest in the image, the analyzed phasor plot is referred as segmented phasor plot.

### Analysis of average points and standard deviation of segmented phasor clusters

A total of at least 15 independent regions from FLIM or spectral images of cell/tissue samples (n = 3) were selected. The regions were selected because they mapped to a specific phasor cluster of interest. The average point of the phasor cluster corresponding to the selected regions was calculated using the reciprocal phasor plot tool of SimFCS (Laboratory of Fluorescence Dynamics; UC Irvine) and the SD was determined for each group of average points. A scatter plot of the average points and the corresponding SD for each sample population was generated. Statistical significance testing was performed between 2 sample populations of interest using t test (P < 0.05 significance level). All statistical analyses were performed using IBM SPSS Statistics (Armonk, NY, USA).

### 2PM spectral emission and FLIM profiling of fluorophore controls

Prior to examining the fluorescence characteristics of melanocyte cytoplasmic melanins, 2PM FLIM and spectral control profiles of melanins with various levels of pigmentation and choroid fluorophores were identified at 780 nm (Table 2). Although these controls do not exactly replicate the tissue sample microenvironment completely, they do provide a frame of reference for intracellular melanin lifetimes and spectra. It is impossible to obtain controls that directly and completely replicate the human choroid microenvironment, given its complexity. The strategy of using human hair with different pigmentation (eumelanin-enriched black hair and pheomelanin-enriched red hair) as biological controls has been employed previously^25,71^. Further, this also provided a method to test the phasor analysis. Melanins in human hair are localized in the hair cortex^44^. The 2PM spectral and FLIM image of the hair cortex and core were acquired at the depth of 19 µm from observing the surface keratin structure.

**Table 2:** 2PM FLIM and spectral profiling of fluorophore controls.

Fluorophore control	Fluorophore source/s	Fluorophore preparation
Dark pigmented eumelanin-enriched melanins	Dark synthetic melanin solution (1 g/ml; Sigma-Aldrich)	10 μl fluorophore solution - fluoroDish (World Precision Instruments)
	Dark brown human hair	Mounted in 100% glycerol
	Dark pigmented central choroid flatmount	Mounted in 100% glycerol
Medium pigmented eumelanin-pheomelanin mixture	Red-brown pigmented bird feather	Mounted in 100% glycerol
Light pigmented pheomelanin-enriched melanins	Red human hair	Mounted in 100% glycerol
	Amelanotic human uveal melanoma cross-section	Mounted in 100% glycerol
	Lightly pigmented central human choroid flatmount (19 weeks’ gestation)	Mounted in 100% glycerol
ECM (collagen I and elastin)	Bovine collagen I (Sigma-Aldrich)	10 μl fluorophore solution - fluoroDish (World Precision Instruments)
	Bovine elastin (Sigma-Aldrich)	10 μl fluorophore solution - fluoroDish (World Precision Instruments)
	Segmented ECM – central choroid flatmount	Mounted in 100% glycerol
Hoechst 33342-stained nucleic DNA	DNA - Hoechst 33342 (1:20,000)-stained nuclei of central choroid flatmount	Mounted in 100% glycerol

### 2PM FLIM and spectral profiling of intracellular melanin in HCM cells

2PM FLIM and spectral imaging profile of label-free and fixed HCMs *in vitro* (n = 3, age: 59–77 years) with heterogeneous pigmentation (very dark to very light) were obtained at 780 nm.

The acquired FLIM and spectral phasor distributions were segmented by mapping specific phasor clusters to the intracellular melanin content of HCM cells. This segmentation process was repeated with the merged phasor plots derived from HCM cells and the various fluorophore controls (melanins of various pigmentation levels and choroidal fluorophores) to verify the level of melanin pigmentation that has been segmented within the HCM cells.

### 2PM FLIM from intracellular melanin mixture and cytoplasmic NADH

From previous studies, the most prominent endogenous fluorophore present within HCMs is the complex biopolymer melanin, comprised of a mixture of eumelanin and pheomelanin; dark pigmented eumelanin has a short fluorescence lifetime and light pigmented pheomelanin has a long fluorescence lifetime^25^. When the 2PM FLIM phasor distribution was acquired from the ‘very dark to very light’ pigmented HCMs, we expected the resultant phasors to be distributed linearly between phasors mapped to eumelanin and pheomelanin-enriched melanin mixture.

However, all cells including melanocytes contain the ubiquitous endogenous cytoplasmic NADH and cytochrome complex endogenous fluorophores within the mitochondrial inner membrane^34,72^. To verify if the acquired FLIM phasor distribution from the HCM intracellular melanin was distinct from cytoplasmic NADH, we included a cytoplasmic NADH control in the study. Melanin-free HEK293 cells (human embryonic kidney 293 cell line, ATCC) were used as a cytoplasmic NADH control. Label-free HEK293 and HCM cells were excited at 740 nm, the maximal excitation wavelength for NADH^42^.

2PM FLIM phasor plots from fixed HCMs, at NADH-optimized 740 nm excitation and at the optimal excitation for intracellular melanin (780 nm), were merged and examined for phasor distribution variation. In addition, 2PM FLIM phasor plots from unfixed and fixed HEK293 cells were merged and examined for the effect of fixation on cytoplasmic NADH. HEK293 cells were fixed with 2% paraformaldehyde (Sigma-Aldrich) in PBS (pH 7.4) for 30 minutes, followed by PBS rinses. Finally, the 2PM FLIM phasor plots measured for fixed HEK293 and HCM cells at 740 nm were merged, to verify if the acquired FLIM phasor distribution from intracellular melanin was distinct from cytoplasmic NADH.

### Single point measurements of spectral emission and fluorescence lifetimes from increasing concentration of synthetic melanin

Dark synthetic melanin solution (1 g/ml; Sigma-Aldrich) was prepared to produce a range of melanin concentrations (0.01 mg/ml, 0.025 mg/ml, 0.05 mg/ml, 0.1 mg/ml) in MilliQ water. The preparation of the solubilized synthetic melanin was based on a protocol used by Riesz *et al*.^73^. The pH of the solutions was adjusted using 1 M NaOH to around 11.5 with constant stirring to aid solubility.

Single point measurements of spectral emission and fluorescence lifetime were acquired using the Fluoromax-4C spectrofluorometer Horiba Scientific. For spectral emission single point measurements, synthetic melanin samples (0.01 mg/ml, 0.025 mg/ml, 0.05 mg/ml, 0.1 mg/ml) were excited at 390 nm and spectral emission were collected between 405–700 nm. The fluorescence lifetime single point measurements were acquired at 370 nm excitation, the only laser source for this module.

### Examination of HCM immunofluorescence using 2PM FLIM phasor technique

HCMs were immunolabeled using standard protocols. Chamber slides with HCMs were fixed and rinsed in PBS, incubated in 5% bovine serum albumin (BSA)/PBS for one hour at room temperature, followed by overnight incubation in melanocyte-specific Melan-a Ab3 primary antibody (Cellular localization: melanosome membrane, endoplasmic reticulum, Golgi apparatus, Host: mouse; NeoMarkers, Fremont CA; 2 µg/ml in 1% BSA/PBS) at 4 °C on a shaking table. Following PBS rinses with shaking, antibody localization was visualized with donkey anti-mouse secondary antibody conjugated to Alexa Fluor 594 (Molecular Probes; 1:1000 in 1% BSA/PBS) for 2 hours at room temperature followed by PBS rinses with shaking. Chamber slides they were mounted in 100% glycerol and coverslipped.

Immunolabeled HCMs were examined using a 2PM FLIM phasor plot where phasor distribution from HCM endogenous melanins and the applied antibody conjugated fluorophore, Alexa Fluor 594 were investigated to confirm that the melanocyte-specific immunolabelling was mainly detected from HCMs with light melanin pigmentation.

### Intracellular melanin profile of HCMs

The intracellular melanin histogram profile from the sample image field of HCMs was generated by determining the ratio fraction from each of the image segmented 2PM FLIM (and spectral) phasor clusters mapped to the heterogeneously pigmented intracellular melanin. The intracellular melanin phasor cluster ratio fraction was determined from the following equation:

$$\frac{Number\,of\,image\,pixels\,mapped\,to\,segmented\,FLIM\,or\,spectral\,phasor\,cluster}{Total\,image\,pixels\,within\,sampled\,image\,field}$$

Ratio fraction measurements were performed using the SimFCS Stats function.

### Profiling intracellular melanin of HCMs and surrounding choroidal components

The intracellular melanins contained in label-free and fixed HCMs in choroid flatmounts (n = 8; age: 49–76 years) and tissue cross-sections (n = 3; age: 59–72 years) were examined and profiled applying the acquired 2PM FLIM and spectral emission data and phasor plot segmentation. The fraction percentage of image pixels associated with segmented intracellular melanin (based on the FLIM and spectral phasor plot) per total pixels from the sample field was measured from cultured HCMs, HCMs in flatmounts and cross-sections. This enabled the comparison of the segmented intracellular melanin from both FLIM and spectral phasor plot techniques.

In addition, FLIM and spectral phasor clusters mapping to the other endogenous excited fluorophores within the tissue samples, such as ECM molecules (e.g. collagen and elastin), heme in RBCs, lipofuscin-melanofuscin in the RPE cells, and added nucleic DNA binding stain were segmented and examined. The cell nuclei of flatmounts and cross-sections were stained with Hoechst 33342 to identify the distribution of choroidal cells including HCMs, fibroblasts, macrophages and mast cells^1^.

The effects of different paraformaldehyde fixation times on the fluorescence lifetimes and spectral emission characteristics of intracellular melanin *in situ* were also investigated, and no effect was observed (Supplementary Note 11).

## Supplementary information

Supplementary Information
