# Supplementary material for: Shedding light on melanins within in situ human eye melanocytes using 2-photon microscopy profiling techniques
Source: Sci Rep. 2019 Dec 9;9:18585. doi: 10.1038/s41598-019-54871-y (PMC6901595; doi:10.1038/s41598-019-54871-y)
Supplement: Supplementary file 1 — Supplementary Information [file 41598_2019_54871_MOESM1_ESM.pdf]

Supplementary Information

Shedding light on melanins within *in situ* human eye melanocytes using 2-photon microscopy profiling techniques

Author list: Ephrem Sitiwin[1,2,4*], Michele C. Madigan[1,4], Enrico Gratton[3], Svetlana Cherepanoff[5], Robert Max Conway[4], Renee Whan[2], Alexander Macmillan[2*]

[1]School of Optometry and Vision Science, University of New South Wales, Sydney, NSW 2052, Australia. [2]Biomedical Imaging Facility, University of New South Wales, Sydney, NSW 2052, Australia. [3]Laboratory for Fluorescence Dynamics, Department of Biomedical Engineering, University of California Irvine, USA. [4]Save Sight Institute, University of Sydney, NSW 2000, Australia. [5]St Vincent's Hospital Sydney, NSW 2010, Australia. * Correspondence and requests for materials should be addressed to E.S. (email: e.sitiwin@unsw.edu.au or efroix@gmail.com) or A.M. (email: alex.macmillan@unsw.edu.au).

Supplementary Note S1

2PM of HCMs and surrounding human choroidal tissue *in situ*. Label-free and fixed HCMs in culture and *in situ* (choroidal flatmounts and paraffin-embedded sections) were imaged by brightfield (**Supplementary Figure S1a, S1c, S1e**) and 2PM (**Supplementary Figure S1b, S1d, S1f**). 2-photon excitation was performed at 780 nm, the optimal intracellular melanin excitation (**Supplementary Note S2).** 2PM images were collected in two channels (500 – 550 nm and 575 – 610 nm, with red and green lookup tables applied respectively) that when overlaid, showed colocalizing pixels colored yellow.

The morphology and distribution of HCMs in cell culture (**Supplementary Figure S1b**), tissue flatmount (**Supplementary Figure S1d**) and cross-section (**Supplementary Figure S1f**) were clearly and consistently identified using endogenous melanin autofluorescence. The cytoplasmic melanosomes within the HCMs can be easily identified as dark spots (**Supplementary Figure S1a, S1d, S1e**). The fluorescence emission detected from dark pigmented eumelanin enriched melanocyte cells (**D**) was uniformly green (575 – 610 nm emission). The light pigmented pheomelanin dominant melanocytes (**L**) showed mostly yellow fluorescence (mainly 500 – 550 nm emission). The mixed pigmented melanocytes (**Mx**), with mixed eumelanin and pheomelanin, showed both green and yellow fluorescence emission.

The 780 nm excitation wavelength provided optimal intracellular melanin excitation, however the fluorescence emission from surrounding extracellular matrix (ECM), red blood cells (RBCs) within blood vessels, RPE and Hoechst 33342-stained nuclei (within HCMs and other choroidal cells) could also be clearly visualized. An image of a human choroid cross-section stained with hematoxylin and eosin (H&E) (systematically used in diagnostic histopathology [1]) is included for comparison (**Supplementary Figure S1g**). The morphology and distribution of HCMs and the surrounding choroidal tissue components identified with 2PM can be seen with H&E staining.

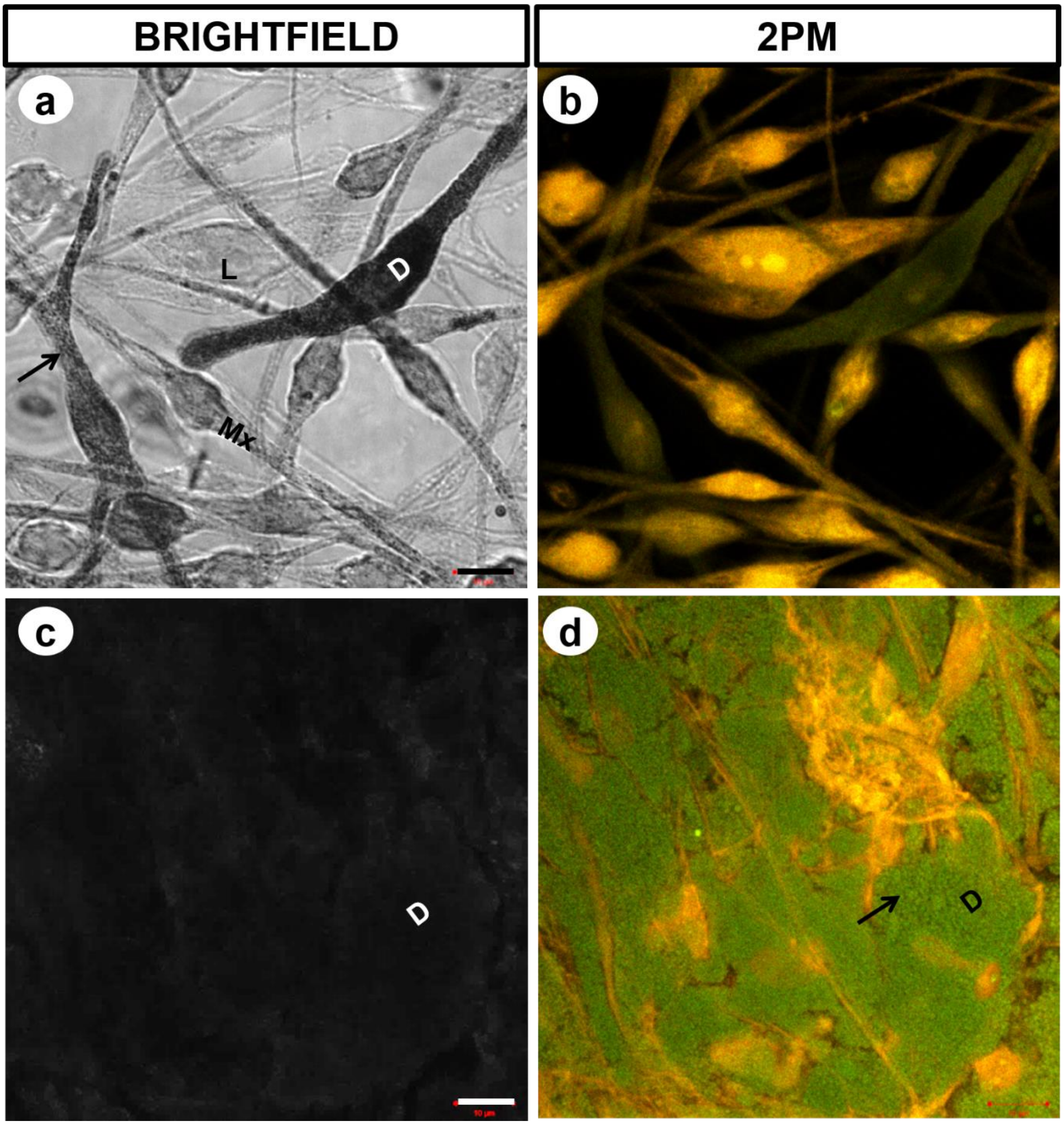
BRIGHTFIELD
2PM
a
b
c
d
L
D
Mx
D
D

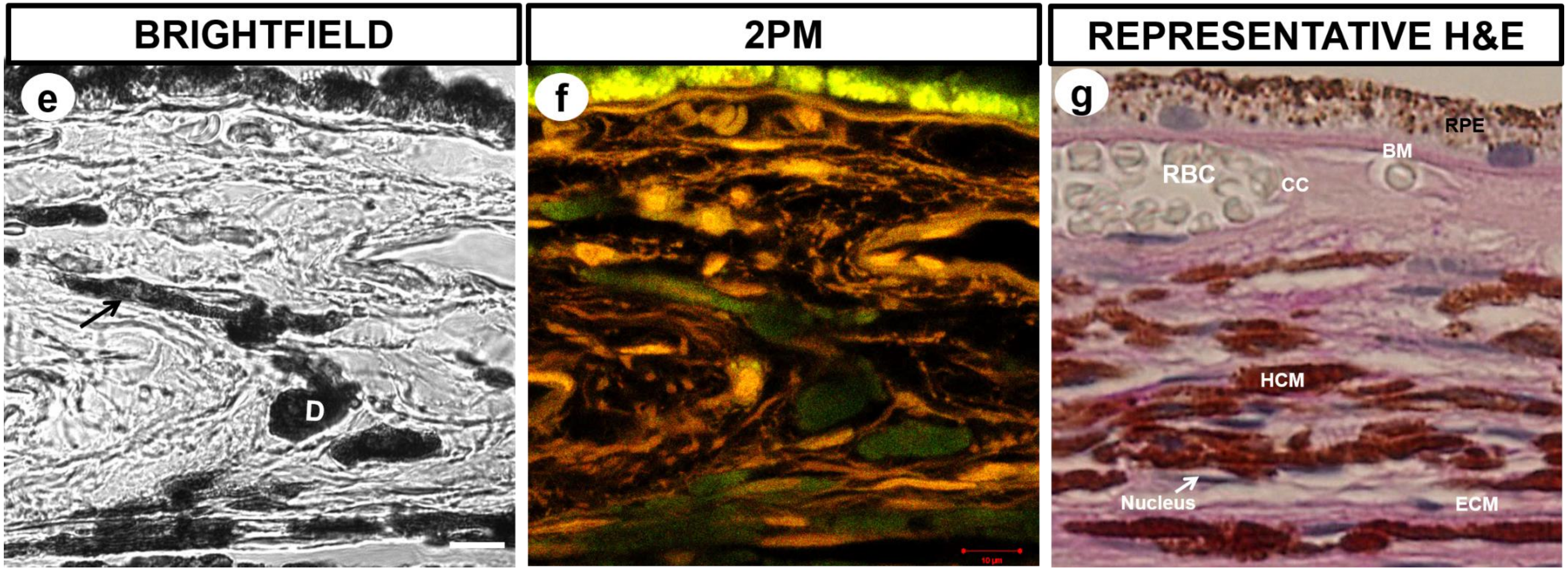

Supplementary Figure S1: HCM morphology and distribution *in situ*. (a & b) Label-free and fixed HCMs *in vitro*, (c & d) human choroid flatmounts, (e & f) paraffin cross-sections were imaged using 2PM, (g) representative H&E stained human choroid cross-section. Images (a, c & e) were acquired by brightfield. Images (b, d & f) were acquired using 2PM. Endogenous melanin fluorophores were excited at 780 nm and fluorescence emission was collected by 2 non-descanned bandpass filters: BP500 – 550 nm (red lookup table applied) and BP575 – 610 nm (green lookup table applied). (a) HCMs have heterogeneous pigmentation: dark (**D**), mixed (**Mx**), light (**L**). Melanosomes (dark spots, arrow) can be seen within the cells. (b) The fluorescence emission detected from darkly pigmented eumelanin enriched HCMs (**D**) were uniformly green (575 – 610 nm emission). The light pigmented pheomelanin dominant HCMs showed mostly yellow fluorescence (mainly 500 – 550 nm emission). The mixed pigmented HCMs, with mixed eumelanin and pheomelanin, showed both green and yellow fluorescence emission. (c)

HCMs in flatmount tissue have dark pigmentation (**D**). (d) Melanosomes (dark spots, arrow) can be seen within the cells. The fluorescence emission detected from dark pigmented HCMs (**D**) was uniformly green (575 – 610 nm emission). (e) HCMs in cross-section tissue have dark pigmentation (**D**). Melanosomes (dark spots, arrow) can be seen within the cells. (f) The fluorescence emission detected from dark pigmented melanocyte HCMs (**D**) was uniformly green (575 – 610 nm emission). HCM morphology, distribution and the surrounding choroidal components identified in (f) were identified in the representative H&E stained choroid section (g) for comparison. **D** = Dark pigmented HCM; **L** = Light pigmented HCM; **Mx** = Mixed pigmented HCM; Arrow = melanosome structures; RBC = red blood cell; cc = choriocapillaris; ECM = extracellular matrix; BM = Bruch's membrane; HCM = human choroidal melanocyte; RPE = retinal pigment epithelium. Scale bar = 10μm.

Supplementary Note S2

Optimal 2PM laser excitation wavelength for intracellular melanins. It was initially necessary to identify the optimal 2-photon pulsed laser wavelength to excite the cytoplasmic melanin mixture within label-free and fixed heterogenous pigmented HCMs because melanin is a low quantum yield fluorophore [2, 3, 4, 5]. To ensure that we collected fluorescence signal mostly from intracellular melanins, there should be limited excitation from other endogenous fluorophores present in HCMs and the choroidal microenvironment. NADH and NADPH, ubiquitous source of autofluorescence in all cells, are typically excited at 740 nm for optimal excitation [6, 7]. In the choroidal ECM, collagens are optimally excited at 730 nm [8, 9] and elastins at 750 nm [10].

This was identified by performing a series of 2-photon lambda (ʎ) emission spectral scans and FLIM measurements between 700 and 900 nm. The results from the emission spectral scans and derived phasor plots showed two excitation wavelengths (λex) that produced the highest mean peak emission intensity (0.44 a.u.): 780nm (Ex780nm) and 830nm (Ex830nm) (**Supplementary Figure S2a**). Intracellular melanin excitation at 780nm provided a more consistent emission intensity peak based on the smaller standard deviation for the measured emission intensity data ($0.4SD_{780nm}$ vs. $0.56SD_{830nm}$).

2PM FLIM phasor distribution derived from the HCM-localized intracellular melanin mixture was also examined between 700 and 900 nm (**Supplementary Figure S2b**). The derived phasors were consistently linear distributed when excited between 740 and 900 nm. When the FLIM phasor plots associated with Ex780nm and Ex830nm were examined closely, intracellular melanin excitation at Ex780nm was more comprehensive than Ex830nm at deriving a phasor distribution mapping to all regions of the heterogeneous pigmented HCMs, particularly phasors associated with the darker pigmented HCMs. Based on these findings and the more consistent fluorescence emission peak, we elected to excite the intracellular melanin mixture within HCMs at 780 nm in the current study.

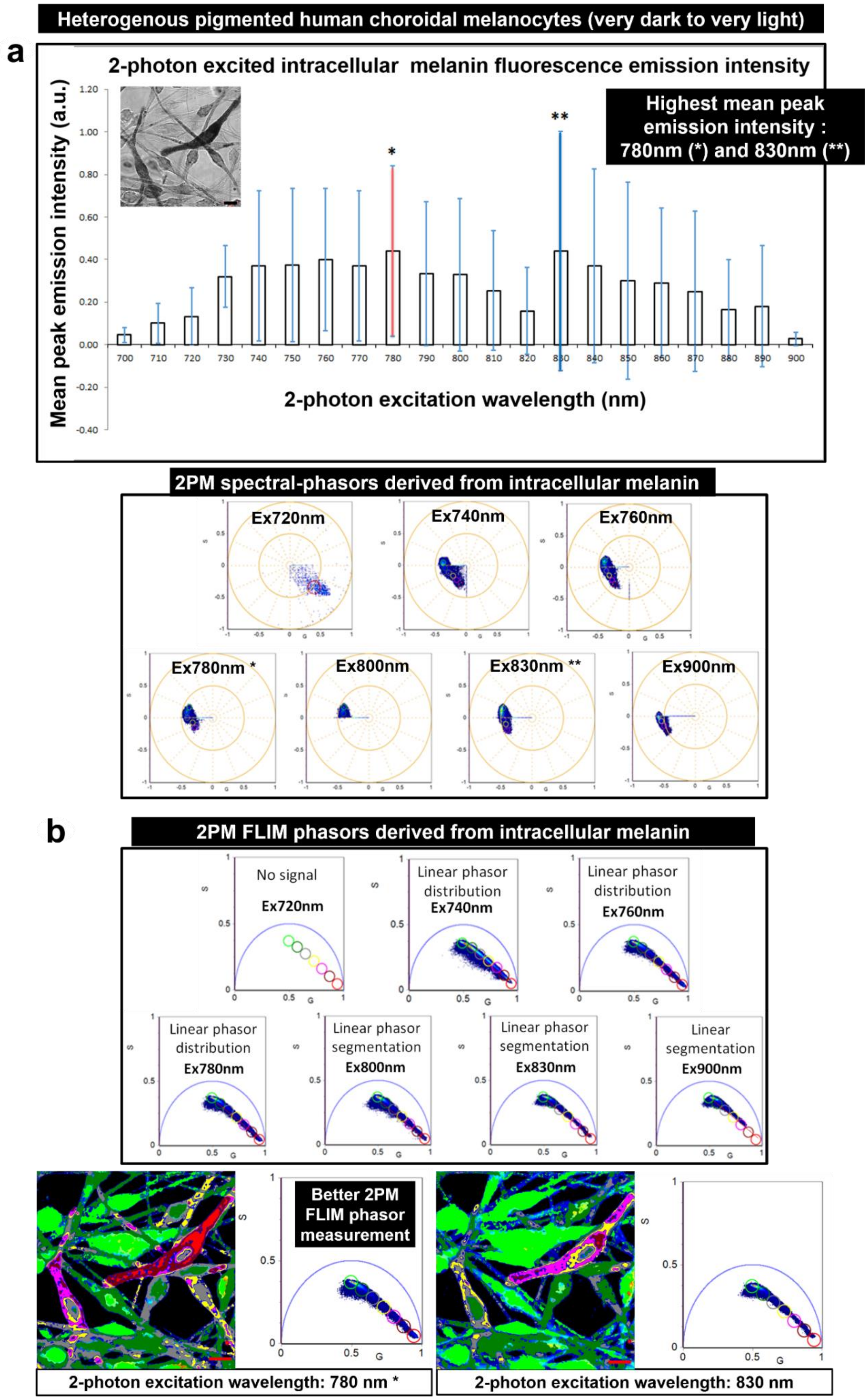
Heterogenous pigmented human choroidal melanocytes (very dark to very light)
a
2-photon excited intracellular melanin fluorescence emission intensity
Mean peak emission intensity (a.u.)
Highest mean peak emission intensity : 780nm (*) and 830nm (**)
2-photon excitation wavelength (nm)
2PM spectral-phasors derived from intracellular melanin
Ex720nm
Ex740nm
Ex760nm
Ex780nm *
Ex800nm
Ex830nm **
Ex900nm
b
2PM FLIM phasors derived from intracellular melanin
No signal
Ex720nm
Linear phasor distribution
Ex740nm
Linear phasor distribution
Ex760nm
Linear phasor distribution
Ex780nm
Linear phasor segmentation
Ex800nm
Linear phasor segmentation
Ex830nm
Linear segmentation
Ex900nm
Better 2PM FLIM phasor measurement
2-photon excitation wavelength: 780 nm *
2-photon excitation wavelength: 830 nm

Supplementary Figure S2: Optimal 2PM laser excitation wavelength (λ_{ex}) for intracellular melanin. (a) The results from the 2-photon ʎ emission scans and derived phasor plots of label-free and fixed HCMs (n = 3) excited between 700 and 900 nm showed that the intracellular melanin mixture was maximally excited at 780 nm and 830 nm with a peak emission intensity of 0.44 a.u. However, intracellular melanin excitation at 780 nm provided a smaller standard deviation compared to the 830 nm excitation wavelength ($0.4SD_{780nm}$ vs $0.56SD_{830nm}$). (b) 2-photon FLIM phasor plots derived from the HCM-localized intracellular melanin mixture measured between 700 and 900 nm. The melanin-mapped phasors were consistently linear distributed across the different wavelengths, but the phasor distribution associated with the 780 nm excitation wavelength provided better mapping to all regions of the heterogenous level of melanin pigmentation compared to Ex830nm. G = X coordinate of phasor transform ('real' unitless phasor component), S = Y coordinate of phasor transform ('imaginary' unitless phasor component), Scale bar = 10µm.

Supplementary Note 1

Spectral phasor profiling of fluorophore controls. Spectral phasor profiles of various fluorophore controls were obtained at 780 nm. The fluorescence emission measured from the fluorophore controls displayed overlapping spectra (**Supplementary Figure 1**). Fluorescence emission was also acquired from other fluorophores within the human choroid tissue. The peak wavelengths of the spectral phasor center of mass related to these fluorophores were determined: eumelanin-enriched melanins in human dark brown hair cortex (z = 19 µm) and human choroid tissue (610 - 620 nm), pheomelanin-enriched melanins in human red hair cortex (z = 19 µm) and human choroid tissue (565 – 589 nm), endogenous ECM from human choroid tissue (554 to 566 nm), porphyrin complex (heme) in RBCs (PC; 612 nm), nucleic DNA binding stain (Hoechst 33342; 540 nm) and hair keratin (560 nm). The phasor distributions from the fluorophore controls were merged (**Supplementary Figure 1e**) and used as controls for subsequent 2PM spectral phasor segmentations of intracellular melanin and surrounding choroidal components.

2PM SPECTRAL PHASOR PROFILES OF FLUOROPHORE CONTROLS

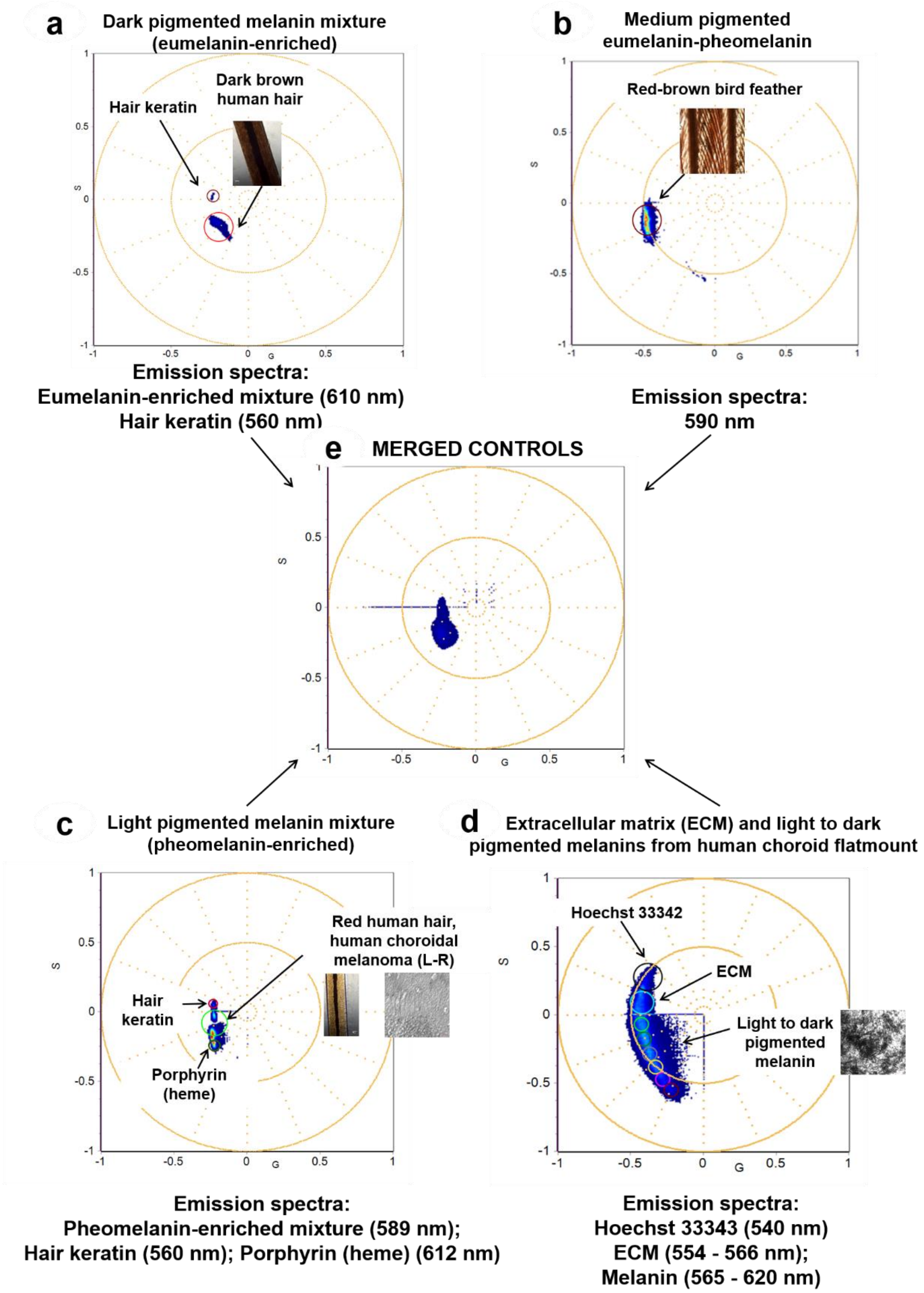

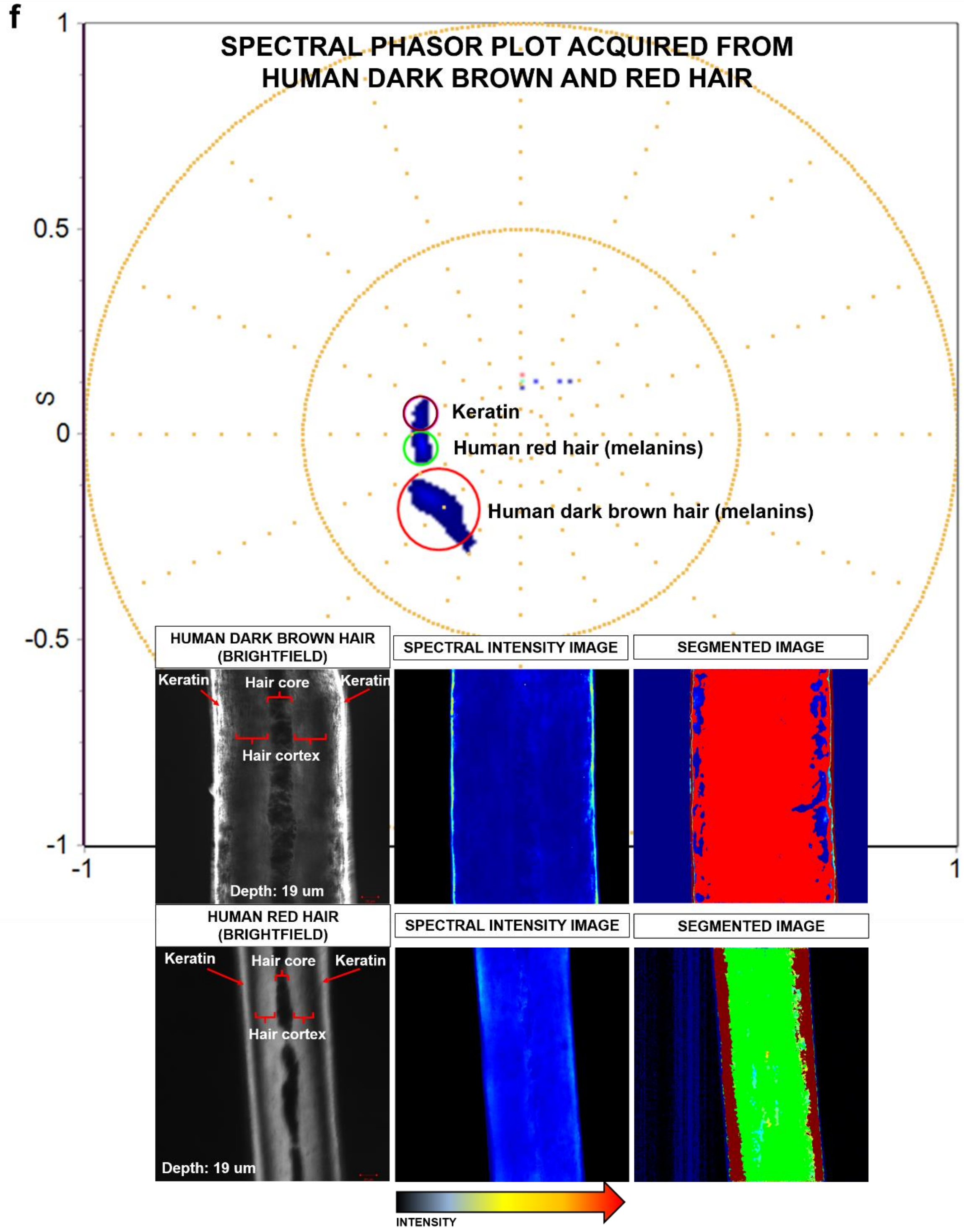

Supplementary Figure 1: 2PM spectral phasor profiling of fluorophore controls. Spectral phasor distribution profiles of various fluorophores used as controls for 2PM spectral phasor segmentations of intracellular melanin and surrounding choroidal components. (a) Dark brown human hair (melanin pigmentation: 'dark' to 'very dark'; eumelanin-enriched);

fluorescence emission spectra red-shifted 610 nm. (b) Red-brown pigmented bird feather (melanin pigmentation: 'medium'; medium mixture of eumelanin and pheomelanin); fluorescence emission spectra 590 nm. (c) Red human hair and human choroidal melanoma (melanin pigmentation: 'mixed' to 'very light'; pheomelanin-enriched); fluorescence emission spectra 589 nm. (d) Light to dark pigmented human choroid tissue; fluorescence emission spectra from 565 nm (pheomelanin-enriched melanins) to 620 nm (eumelanin-enriched melanins). Endogenous ECM from human choroid; fluorescence emission spectra from 554 to 566 nm. (e) The resultant phasor plots were merged and used as a control in subsequent spectral phasor segmentation of HCM cells and human choroid tissue. (f) Merged spectral phasor clusters mapped to eumelanin-enriched melanins in human dark brown hair cortex (z = 19 µm), pheomelanin-enriched melanins in human red hair cortex (z = 19 µm) and hair keratin. G = X coordinate of phasor transform (‘real’ unitless phasor component), S = Y coordinate of phasor transform (‘imaginary’ unitless phasor component).

Supplementary Note 2

Comparing spectral shift identified between dark and light pigmented HCMs and melanin concentration dependent spectral shift. The spectral single point measurements of increasing concentrations of dark synthetic melanin (0.01 mg/ml, 0.025 mg/ml, 0.05 mg/ml, 0.1 mg/ml) showed a spectral shift of around 10 nm, between the lowest (0.01 mg/ml) and the highest (0.1 mg/ml) concentrations (**Supplementary Figure 2a**). The same magnitude of spectral shift between 0.01 mg/ml and 0.05 mg/ml dark synthetic melanin concentrations was previously reported by Riesz et al. (2005) [11].

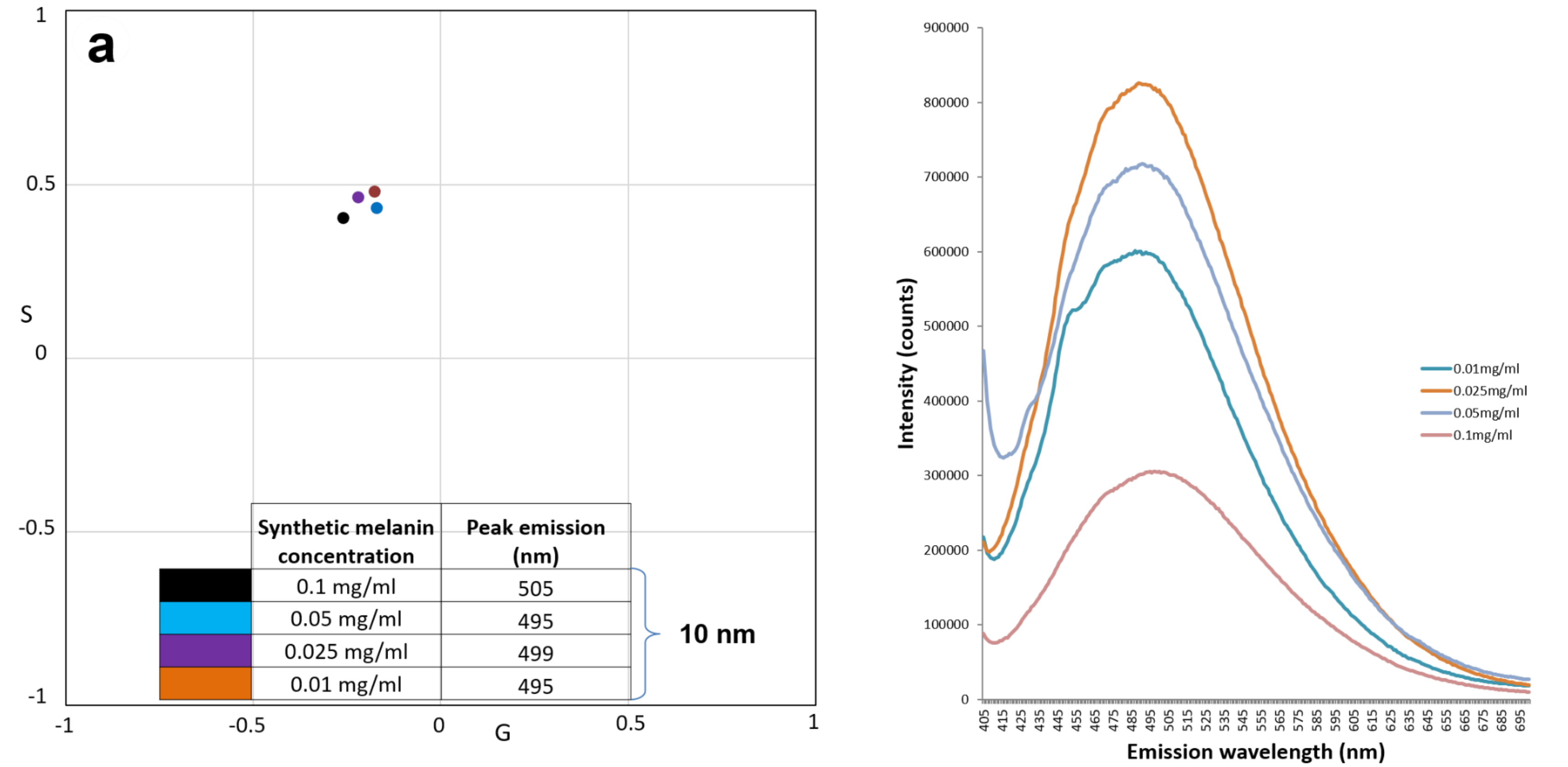

Supplementary Figure 2a: Single point measurements of fluorescence lifetimes from increasing concentration of synthetic melanin. The spectral single point measurements of increasing concentrations of dark synthetic melanin (0.01 mg/ml, 0.025 mg/ml, 0.05 mg/ml, 0.1 mg/ml) showed a spectral shift of around 10 nm, between the lowest (0.01 mg/ml) and the highest (0.1 mg/ml) concentrations. G = X coordinate of phasor transform ('real' unitless phasor component), S = Y coordinate of phasor transform ('imaginary' unitless phasor component).

The fluorescence emission spectra acquired from dark to light pigmented HCMs excited using 2-photon laser at 780 nm showed a spectral shift beyond the melanin concentration dependent spread of 10 nm (**Supplementary Figure 2b**). The peak wavelength of the spectral phasor cluster's center of mass mapped to dark eumelanin-enriched melanocytes was identified at around 635 nm; for light pigmented, pheomelanin-enriched melanocytes, this was identified at around 589 nm. The shift in peak emission identified between dark eumelanin-enriched and light pheomelanin-enriched melanocytes was thus around 45 nm, a wider spread than expected if this was a melanin concentration dependent shift.

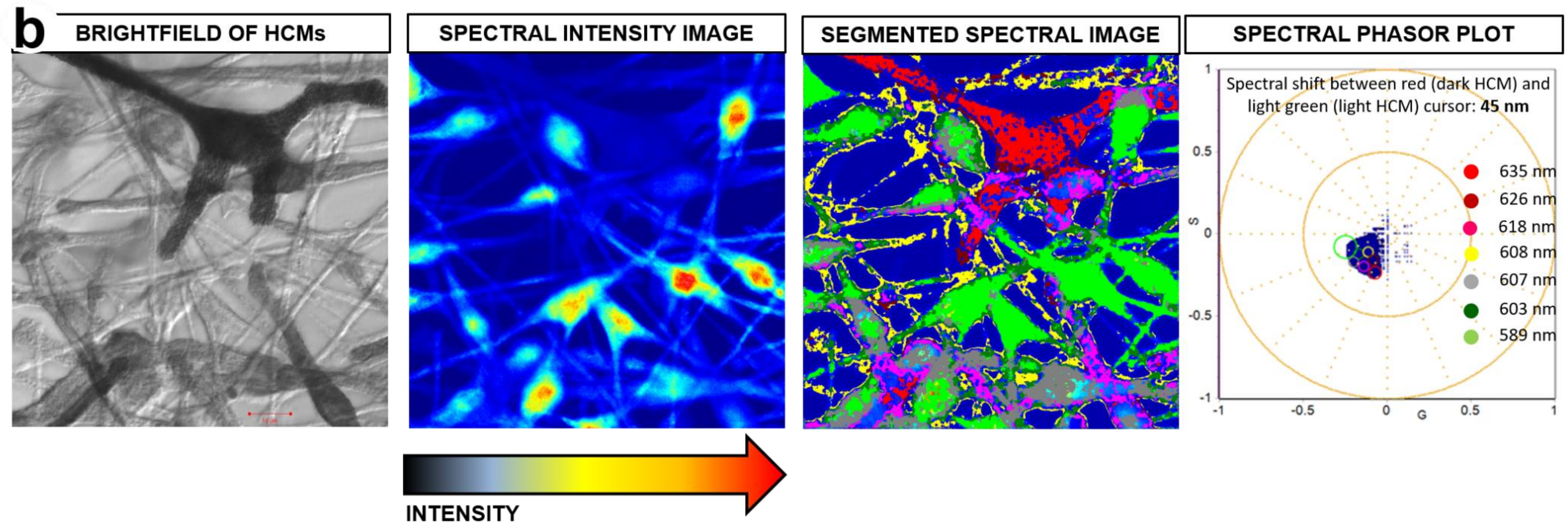

Supplementary Figure 2b: The shift in peak emission identified between dark eumelanin-enriched and light pheomelanin-enriched melanocytes was around 45 nm, a wider spread than the expected melanin concentration dependent shift of around 10 nm. The 2PM spectral signal from HCM cells was improved by increasing acquisition bin size from 8.9 nm to 26.8 nm. However, this results in a three-fold decrease in spectral resolution and therefore, would produce poorer segmentation of closely emitting species. Therefore, the bin size for the 2PM spectral acquisition was maintained at 8.9 nm. G = X coordinate of phasor transform ('real' unitless phasor component), S = Y coordinate of phasor transform ('imaginary' unitless phasor component).

Further to this, we also acquired fluorescence lifetime single point measurements of synthetic melanin solutions with the same increasing concentration as the spectral single point measurements. No change in fluorescence lifetime evident (**Supplementary Figure 2c**).

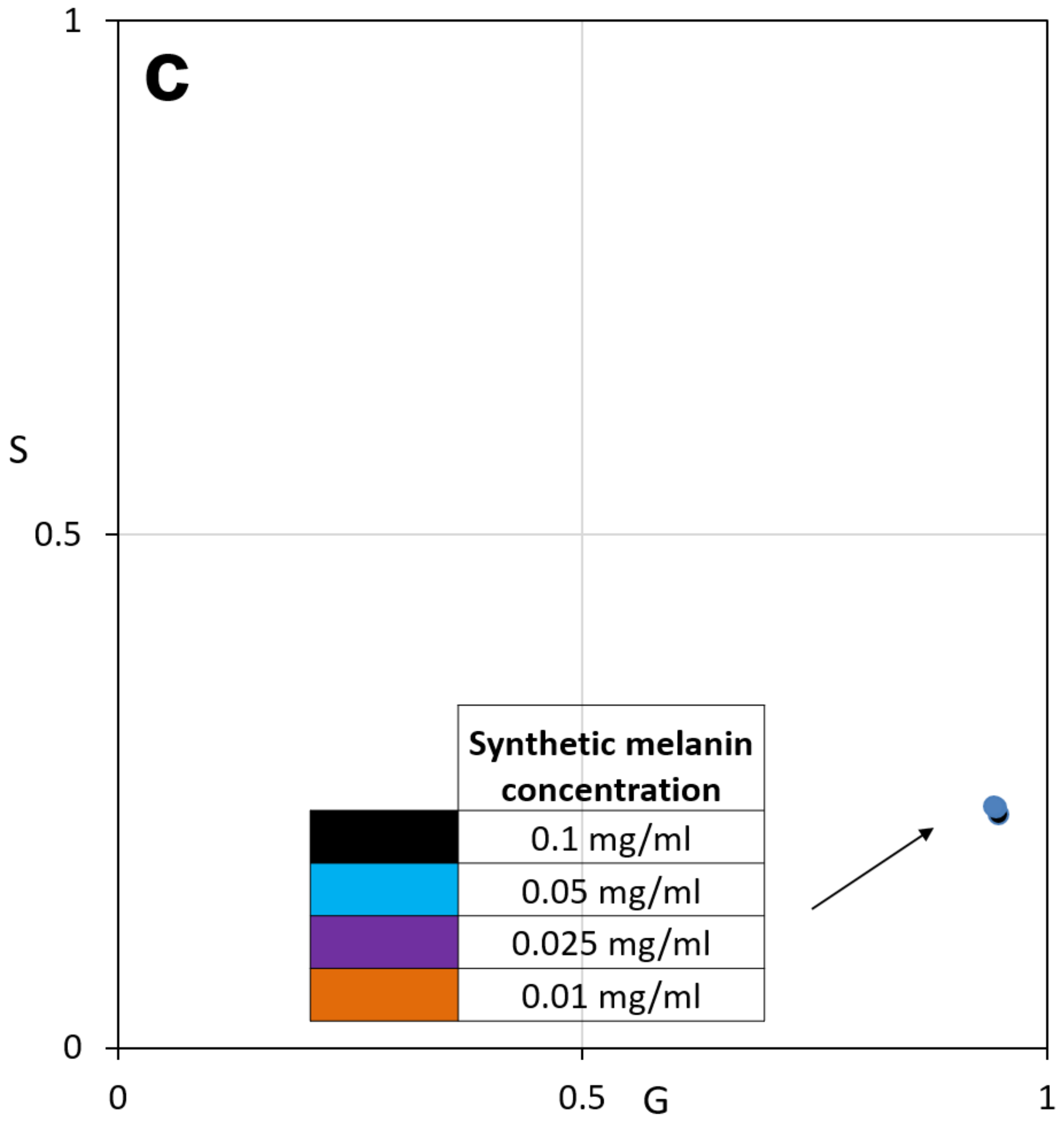

Supplementary Figure 2c: The fluorescence lifetime single point measurements of synthetic melanin solutions (0.01 mg/ml, 0.025 mg/ml, 0.05 mg/ml, 0.1 mg/ml) showed no evidence of change in lifetimes. G = X coordinate of phasor transform ('real' unitless phasor component), S = Y coordinate of phasor transform ('imaginary' unitless phasor component).

Merged segmented phasor clusters from cultured HCMs and the spectral phasor distributions from the melanin fluorophore controls. When segmented phasor clusters from cultured HCMs were merged with the spectral phasor distributions from the melanin fluorophore controls, clusters **(i – iii)** mapped to the eumelanin-enriched dark brown hair and dark pigmented regions of HCMs *in situ*. Cluster **(iv)** mapped to the medium pigmented red-brown bird feather. Clusters (**v – vii**) mapped to the lightly colored pheomelanin-enriched red hair and amelanotic choroidal melanoma (**Supplementary Figure 2d**).

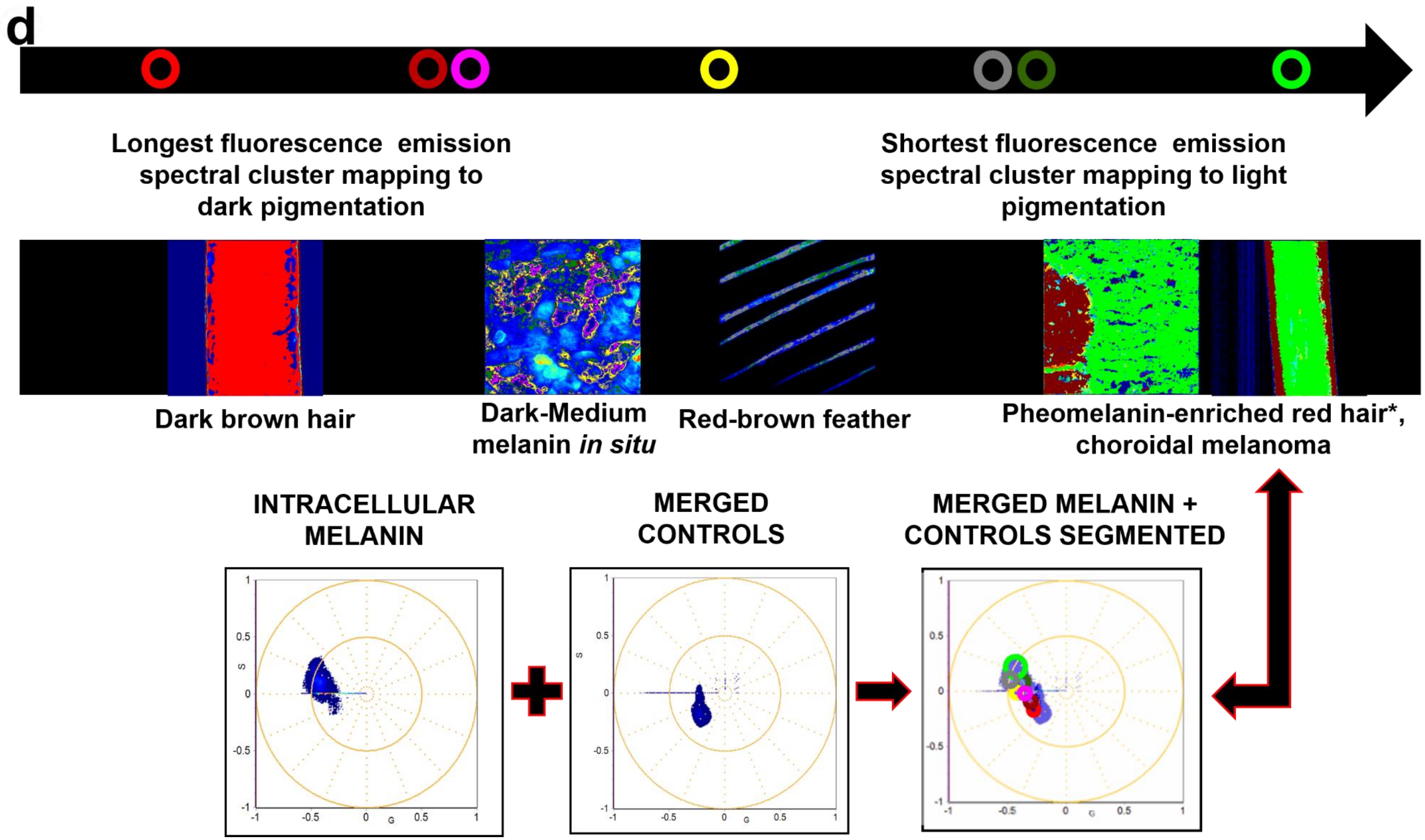

Supplementary Figure 2d: Merging of segmented phasor clusters from cultured HCMs with the spectral phasor distributions from the melanin fluorophore controls. When clusters **(i – vii)** were merged with the phasor distribution measured from the melanin fluorophore

controls, clusters **(i – iii)** mapped to the eumelanin-enriched dark brown hair and the dark pigmented regions of HCMs *in situ*, cluster **(iv)** mapped to the medium pigmented red-brown bird feather, clusters **(v – vii)** mapped to the lightly colored pheomelanin-enriched mixture contained in the red hair and amelanotic choroidal melanoma. G = X coordinate of phasor transform ('real' unitless phasor component), S = Y coordinate of phasor transform ('imaginary' unitless phasor component).

Supplementary Note 3

FLIM phasor profiling of fluorophore controls. FLIM phasor distribution profiles of various fluorophores were used as controls for choroid components (**Supplementary Figure 3**). These were excited at 780 nm and used for subsequent phasor segmentation analysis of HCM-localized melanin and choroid tissue. The merged phasors from the melanin sources (**Supplementary Figure 3a, 3b, 3c, 3d**) displayed a linear phasor distribution. The merged phasor plot of all fluorophore controls (**Supplementary Figure 3e**) was subsequently used as a control for all phasor segmentations of intracellular melanin and surrounding choroidal components.

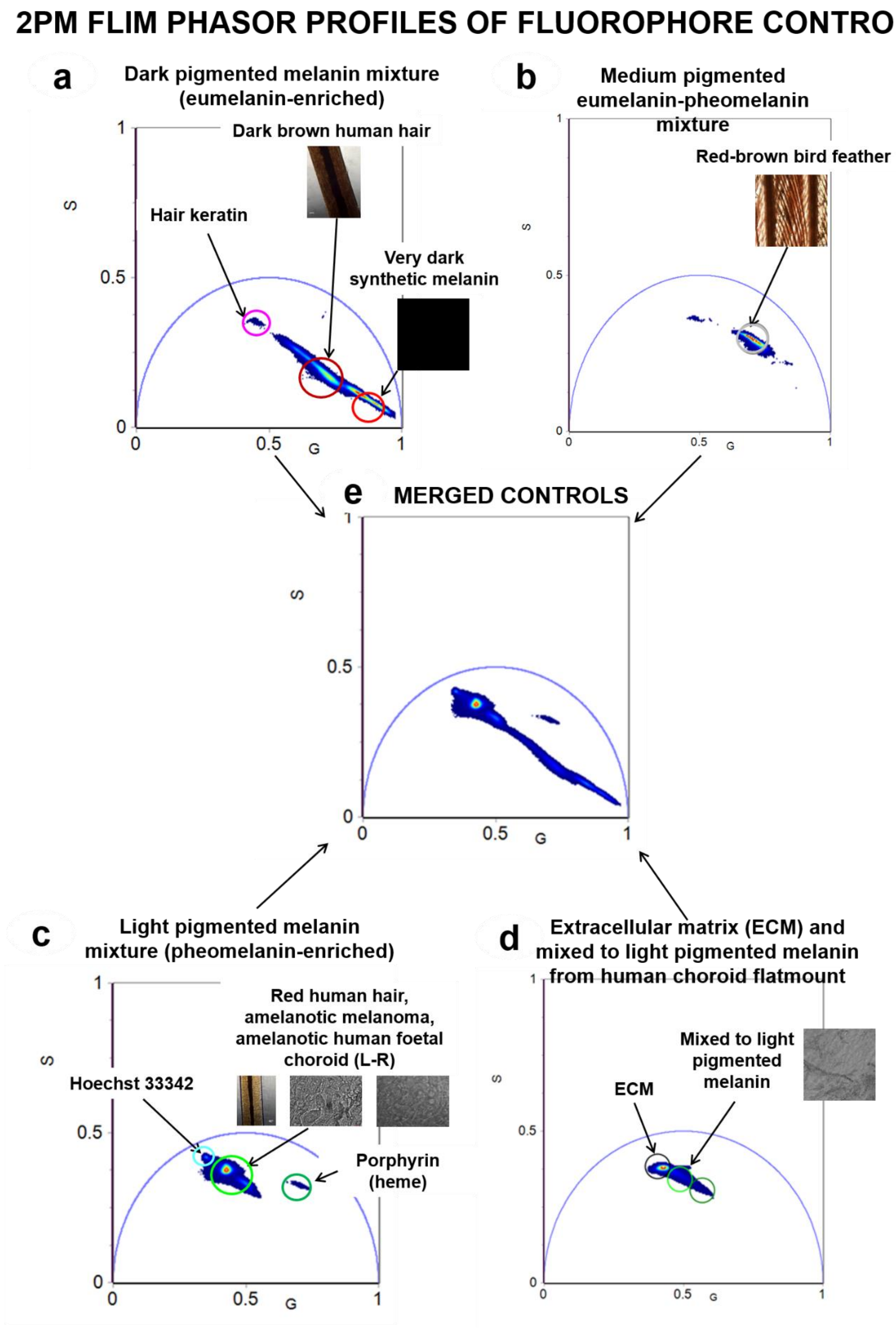

Supplementary Figure 3: 2PM FLIM phasor fingerprints of fluorophore controls. (a to d) FLIM phasor distribution profiles of various control fluorophores. (e) The resultant phasor plots (a to d) were merged and used as a control in subsequent 2PM FLIM phasor

segmentation of HCM cells and human choroid tissue. G = X coordinate of phasor transform (‘real’ unitless phasor component), S = Y coordinate of phasor transform (‘imaginary’ unitless phasor component).

Supplementary Note 4

Merged segmented phasor clusters from cultured HCMs and the FLIM phasor distributions from the melanin fluorophore controls. When the segmented phasor clusters from cultured HCMs were merged with the FLIM phasor distribution measured from the melanin fluorophore controls, phasor clusters (**i – iii**) mapped to dark colored eumelanin-enriched synthetic melanin and dark brown hair. Cluster (**iv**) mapped to the ‘medium’ pigmented red-brown bird feather, consistent with a medium eumelanin/pheomelanin ratio. Clusters (**v – vii**) mapped to the lightly colored pheomelanin-enriched mixture of red hair, amelanotic melanoma and the lightly pigmented human fetal choroid (**Supplementary Figure 4**).

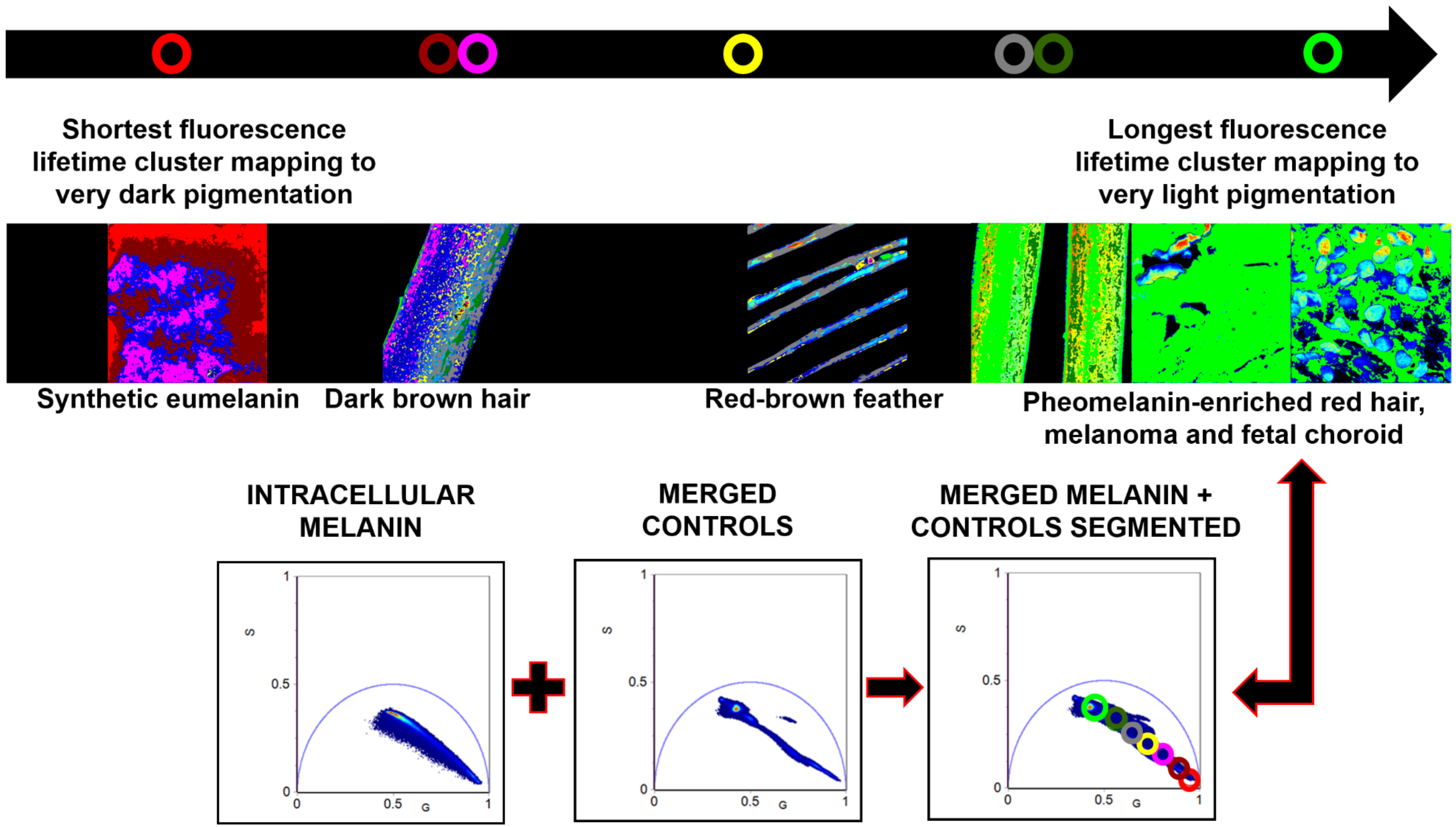

Supplementary Figure 4: Merging of segmented phasor clusters from cultured HCMs with the FLIM phasor distributions from the melanin fluorophore controls. When clusters (**i – vii**) were merged with the FLIM phasor distribution measured from the melanin fluorophore controls, clusters (**i – iii**) mapped to the eumelanin-enriched synthetic melanin and dark brown hair, cluster (**iv**) mapped to the medium pigmented red-

brown bird feather, clusters (**v – vii**) mapped to the lightly colored pheomelanin-enriched mixture contained in the red hair, amelanotic melanoma and the lightly pigmented human fetal choroid. G = X coordinate of phasor transform (‘real’ unitless phasor component), S = Y coordinate of phasor transform (‘imaginary’ unitless phasor component), Scale bar = 10 μm.

Supplementary Note 5

Average lifetimes of melanin controls comparable with average lifetimes measured from HCMs. We fitted the fluorescence decay measured from the eumelanin-enriched dark brown hair, dark pigmented HCM, the pheomelanin-enriched red hair and light pigmented HCM using a two exponentials model (**Supplementary Table 5**). We observed that the average lifetimes (intensity) measured from the dark brown hair (1.7 ± 0.012 ns) comparable with the dark pigmented HCM cell (1.3 ± 0.012 ns). The average lifetimes (intensity) measured from the red hair (2.4 ± 0.0091 ns) comparable with the light pigmented HCM cell (2.1 ± 0.011 ns).

	τ_1 (ns)	α_1 (Counts)	τ_2 (ns)	α_2 (Counts)	T Av Intensity (ns)
**Dark brown hair**	2.2	267900	0.16	1149000	1.7 ± 0.012
**Dark pigmented HCM**	1.7	19200	0.16	63000	1.3 ± 0.012
**Red Hair**	3.1	238100	0.68	457900	2.4 ± 0.0091
**Light pigmented HCM**	2.6	52500	0.55	64450	2.1 ± 0.011

Supplementary Table 5: Fluorescence decay fitting of eumelanin-enriched dark brown hair, dark pigmented HCM, the pheomelanin-enriched red hair and light pigmented HCM using a two exponentials model.

Poorer signal to noise detection in descanned measurements compared to non-descanned measurements. The same field of view of HCM cells were imaged with the same imaging parameters (excitation laser: 780 nm (3.7%), 500 – 550 nm detection wavelength range) using non-descanned and descanned detection. Both images were contrast enhanced to 100 greyscale value. The non-descanned image showed a higher signal to noise compared to the descanned image (**Supplementary Figure 5**). Poorer signal to noise ratio would be the limiting factor to how many fluorophore species can be reliably unmixed using spectral phasor analysis [12].

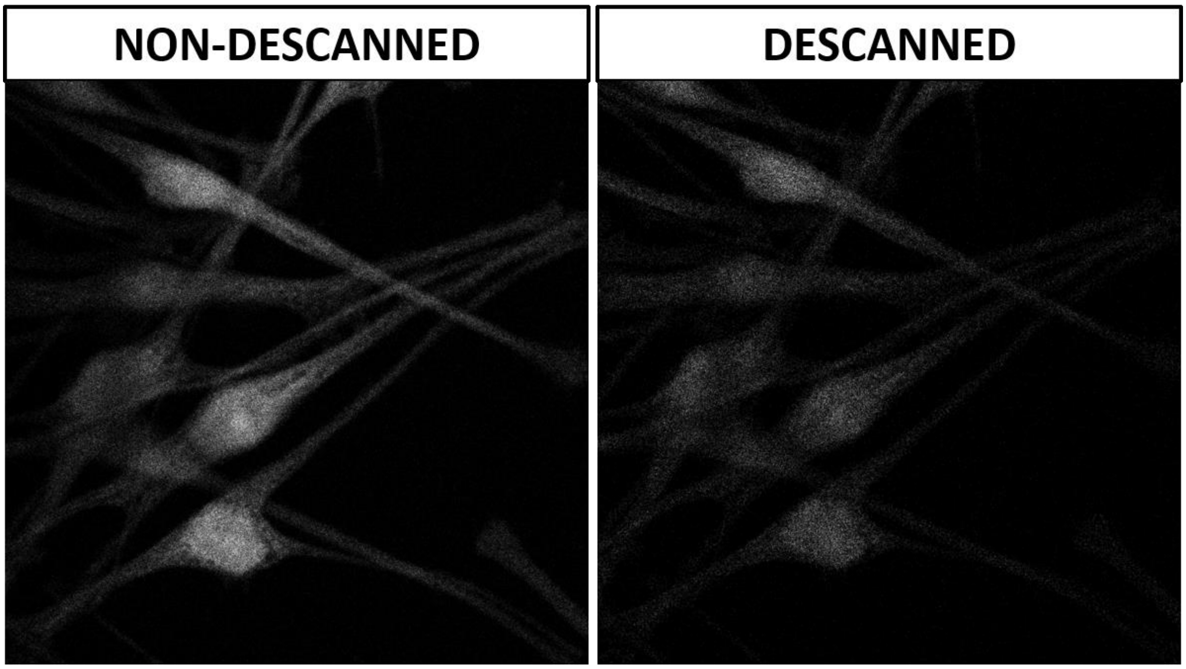

Supplementary Figure 5: Although non-descanned and descanned measurements are not directly comparable, descanned detection suffered from poorer signal to noise ratio even when data were acquired from the same field of view and acquisition parameters.

Supplementary Note 6

Examination of phasor plots from fixed HCM and HEK293 cells excited at 740 nm. Cytoplasmic NADH within the melanin-free HEK293 cells was optimally excited at 740 nm, and 2PM FLIM data acquisition and phasor analysis were also performed at this wavelength on fixed HCMs. Segmented phasor clusters mapping to the light pigmented melanin mixture and cytoplasmic NADH were distinct and positioned close to each other (**Supplementary Figure 6a**). The linear phasor distribution acquired from HCMs at 740 nm (optimal NADH excitation wavelength) and 780 nm (optimal melanin excitation wavelength) overlapped, confirming that the linear phasor distribution was maintained at both 740 nm and 780 nm (**Supplementary Figure 6b**). The FLIM phasor distributions of unfixed and fixed HEK293 cells also overlapped providing evidence that cytoplasmic NADH could be successfully measured in paraformaldehyde-fixed cells (**Supplementary Figure 6c**).

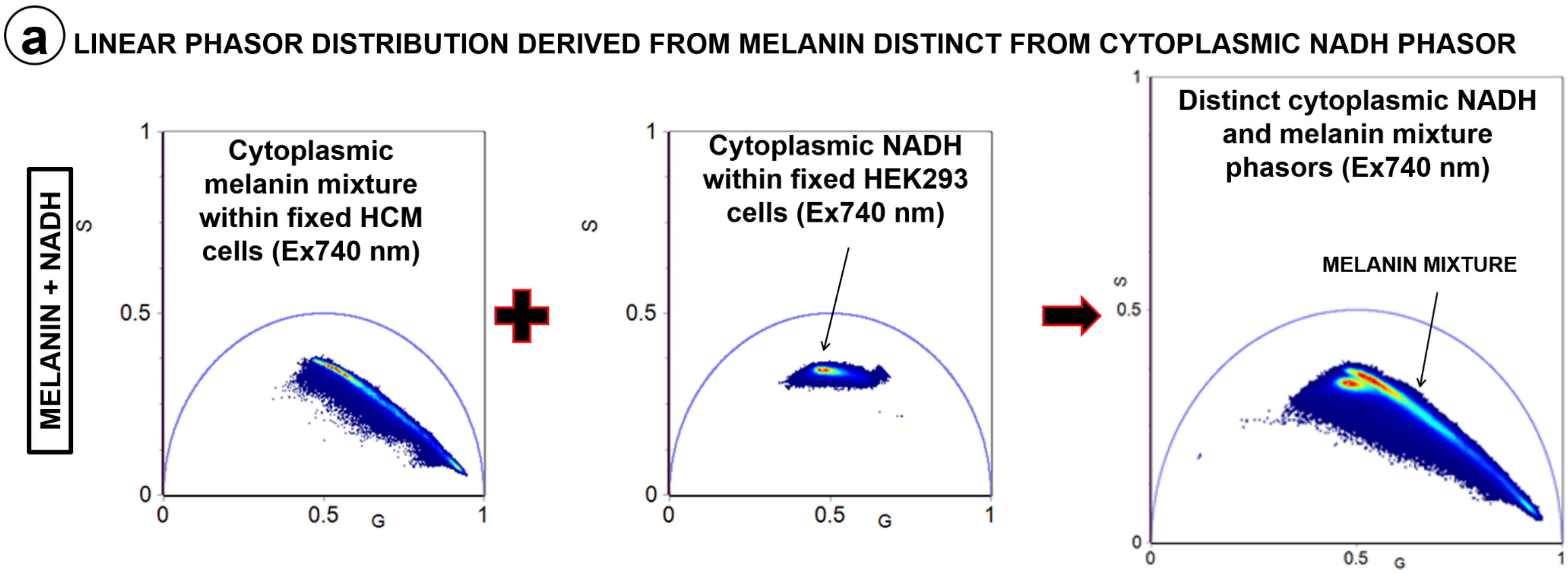

a
LINEAR PHASOR DISTRIBUTION DERIVED FROM MELANIN DISTINCT FROM CYTOPLASMIC NADH PHASOR
MELANIN + NADH
Cytoplasmic melanin mixture within fixed HCM cells (Ex740 nm)
Cytoplasmic NADH within fixed HEK293 cells (Ex740 nm)
Distinct cytoplasmic NADH and melanin mixture phasors (Ex740 nm)
MELANIN MIXTURE
S
G
0
0.5
1

b OVERLAPPING LINEAR PHASOR DISTRIBUTION DERIVED FROM CYTOPLASMIC MELANIN EXCITED AT 740 NM AND 780 NM

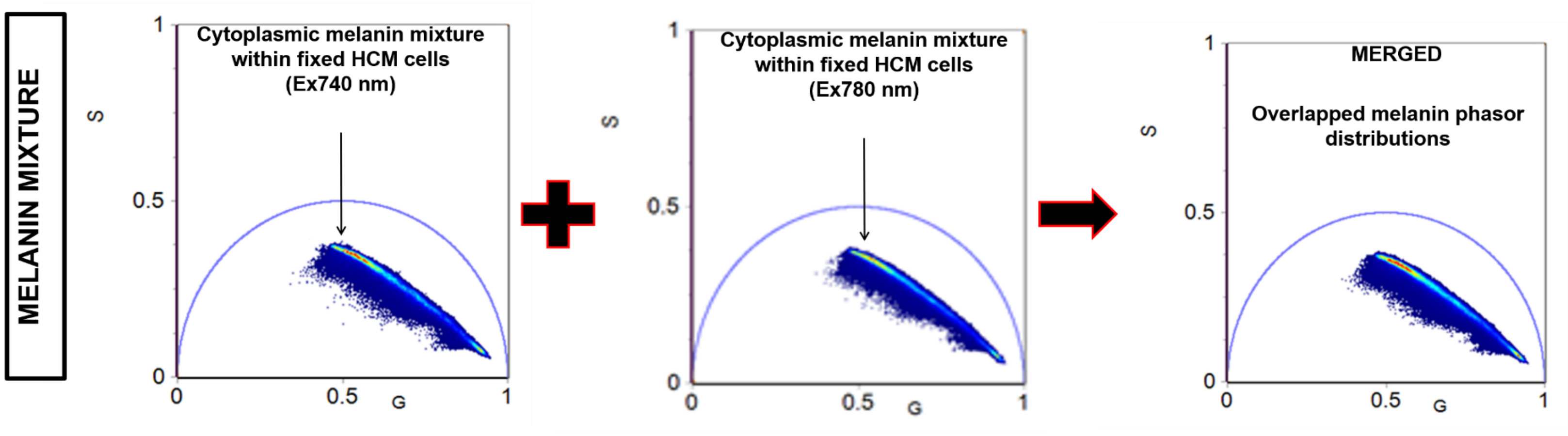

c OVERLAPPING LINEAR PHASOR DISTRIBUTION DERIVED FROM CYTOPLASMIC NADH IN UNFIXED AND FIXED HEK293 CELLS

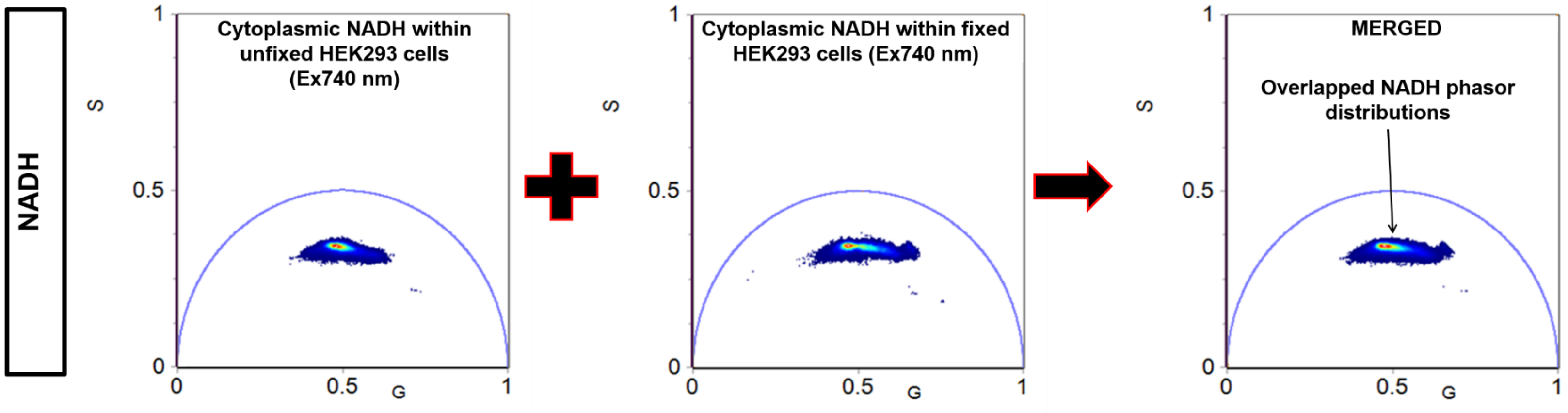

Supplementary Figure 6: 2PM FLIM phasor distribution from heterogeneous pigmented intracellular melanin was distinct but proximal from cytoplasmic NADH phasor distribution. (a) The merged FLIM phasor distribution acquired from fixed HCMs and HEK293 cells showed that distinct phasor distribution was identified from cytoplasmic NADH and HCM intracellular melanins. (b) HCM-derived FLIM phasor distributions for 740 nm and 780 nm overlapped indicating the linear distribution of the phasor distribution was maintained at both excitation wavelengths. (c) FLIM phasor distribution acquired from unfixed and fixed HEK293 cells overlapped, confirming that the fluorescence lifetime characteristics of the ubiquitous cytoplasmic NADH was not affected post-fixation. However, the fixation process may cause the loss of the dynamic properties of the fluorescent biomolecules. G = X coordinate of phasor transform (‘real’ unitless phasor component), S = Y coordinate of phasor transform (‘imaginary’ unitless phasor component).

Supplementary Note 8

Quantitative profile of HCM intracellular melanins based on spectral and FLIM phasors. A quantitative histogram profile of the intracellular melanins within HCM cells of varying pigmentation was generated by identifying the image pixel fraction contributed by each of the image segmented spectral and FLIM phasor clusters (**cluster i – vii; Supplementary Figure 8a).** Spectral and FLIM phasor clusters associated with the 'light' to 'very light' melanin pigmentation featured prominently in the generated profiles. However, the profile generated from image segmented spectral-clusters **(i)** to **(v)** was less well-characterized **(Supplementary Figure 8a)**.

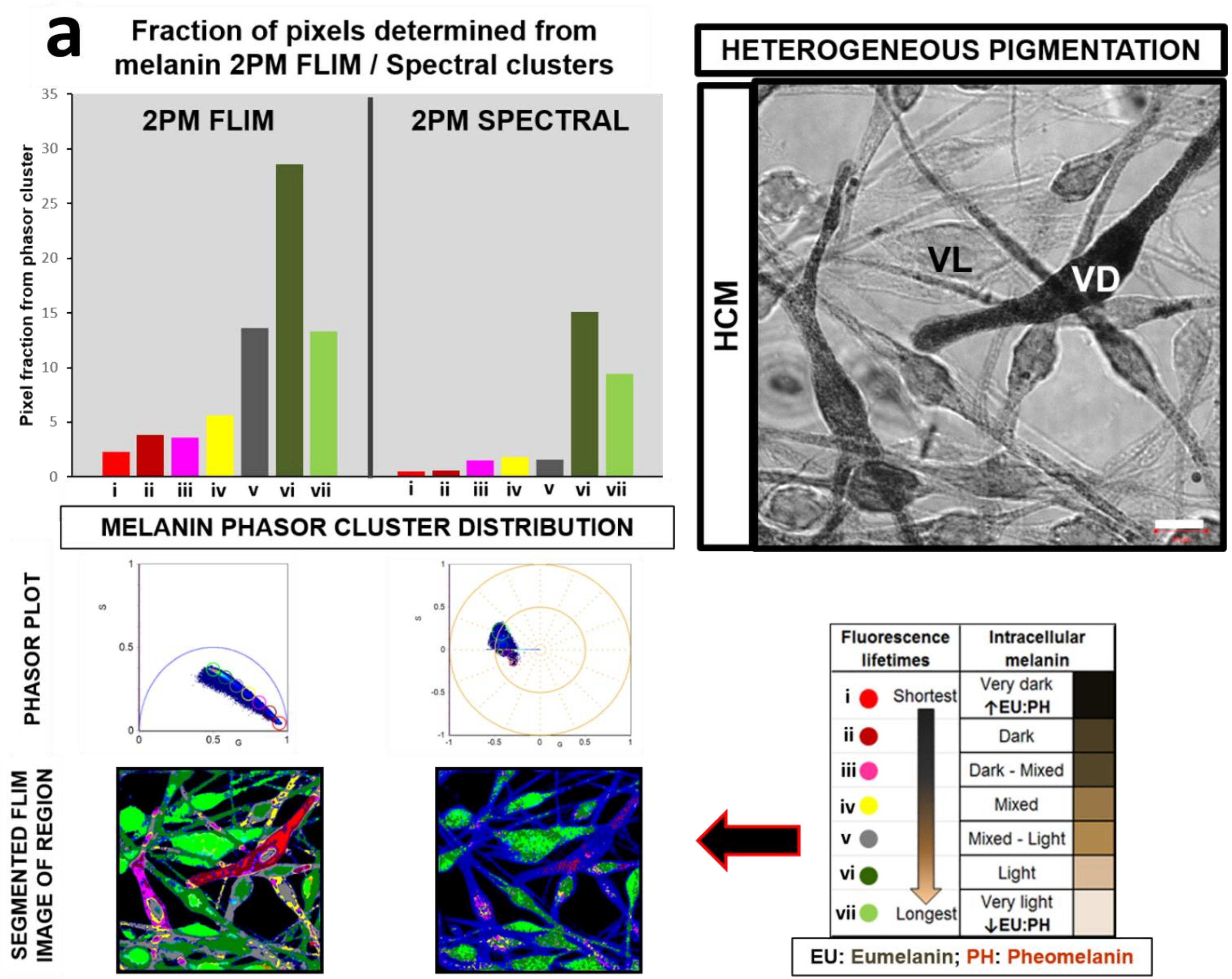
a
Fraction of pixels determined from melanin 2PM FLIM / Spectral clusters
2PM FLIM
2PM SPECTRAL
Pixel fraction from phasor cluster
0
5
10
15
20
25
30
35
i ii iii iv v vi vii
i ii iii iv v vi vii
MELANIN PHASOR CLUSTER DISTRIBUTION
PHASOR PLOT
SEGMENTED FLIM IMAGE OF REGION
HETEROGENEOUS PIGMENTATION
HCM
VL
VD
Fluorescence lifetimes
Intracellular melanin
i Shortest
Very dark ↑EU:PH
ii
Dark
iii
Dark - Mixed
iv
Mixed
v
Mixed - Light
vi
Light
vii Longest
Very light ↓EU:PH
EU: Eumelanin; PH: Pheomelanin

Supplementary Figure 8a: Quantitative histogram profile of melanins in sampled HCM cells. A quantitative profile of the melanins within HCMs was constructed by identifying the image pixel fraction contributed by an image segmented FLIM/spectral phasor cluster mapped to a specific eumelanin/pheomelanin ratio: **cluster i** (eumelanin-enriched melanins) to **cluster vii** (pheomelanin-enriched melanins). The prominent phasor clusters segmented from the sampled HCMs were mapped to 'light' to 'very light' intracellular melanins. Profiling from the darker pigmented cells was less well-characterized for the spectral phasor segmentation. G = X coordinate of phasor transform ('real' unitless phasor component), S = Y coordinate of phasor transform ('imaginary' unitless phasor component). Scale bar = 10μm.

Profiling of intracellular HCM melanins and surrounding choroidal components in tissue cross sections. Intracellular melanins in label-free and fixed HCMs in choroidal tissue cross-sections (**Supplementary Figure 8b**) were examined using FLIM/spectral phasor segmentation and intracellular melanin profiling. FLIM phasor clusters also mapped to surrounding choroidal fluorophores such as ECM (collagens and elastins), RBCs (heme), RPE (lipofuscin, melanofuscin and ubiquitous mitochondrial NADH / cytochrome complex) (**Supplementary Figure 8b**). These were clearly distinct from HCM intracellular melanin phasor clusters. Segmented spectral phasor clusters mapping to HCM intracellular melanins also mapped to other choroidal structures such as ECM due to the overlapping fluorescence emission spectra.

The profiling of intracellular HCM melanins and surrounding choroidal components in all *in situ* specimens is provided in **Supplementary Note 12**.

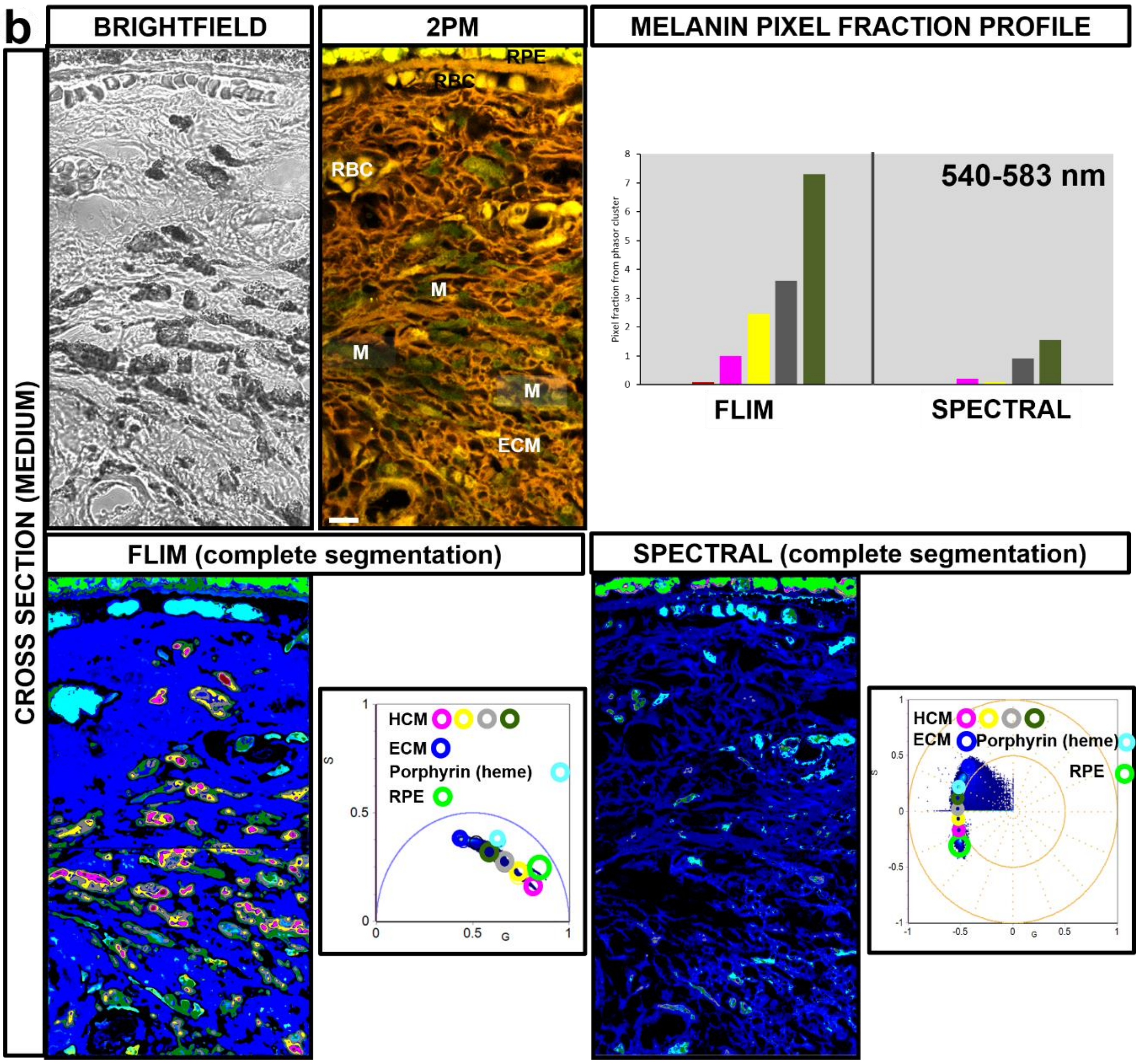

Supplementary Figure 8b: 2PM FLIM/spectral phasor distribution mapping to HCM-localized melanins and surrounding human choroidal section tissue. The segmented FLIM/spectral phasor clusters mapping to 'medium' pigmented HCMs in cross section tissue had medium fluorescence lifetimes / emission spectra: 540 to 583 nm. The FLIM phasor clusters mapped to fluorophores contained in ECM, RBCs, RPE and Hoechst 33342 nucleic DNA binding stain were distinct from the HCM-matched clusters but image segmented spectral phasor clusters showed overlapping emission spectra. M = HCM, ECM = Extracellular matrix, RPE = Retinal pigment epithelium, RBC = Red blood cell, G = X coordinate of phasor transform ('real' unitless phasor component), S = Y coordinate of phasor transform ('imaginary' unitless phasor component), Scale bar = 10μm.

Comparing fraction percentage of image pixels representing intracellular melanin per total sample field image pixels between image segmented FLIM and spectral phasor plot. When the fraction percentage of image pixels representing intracellular melanin per total sample field image pixels was investigated, based on image segmented FLIM and spectral phasor plot (**Supplementary Figure 8c**), the FLIM data (non-descanned mode) gave a higher signal to noise ratio than the spectral emission data: 70% FLIM phasor *versus* 30% Spectral phasor from HCM cells; 75% FLIM phasor *versus* 70% Spectral phasor from HCMs in tissue flatmounts and 10% FLIM phasor *versus* 3% Spectral phasor from HCMs in tissue cross-sections (**Supplementary Figure 8c**).

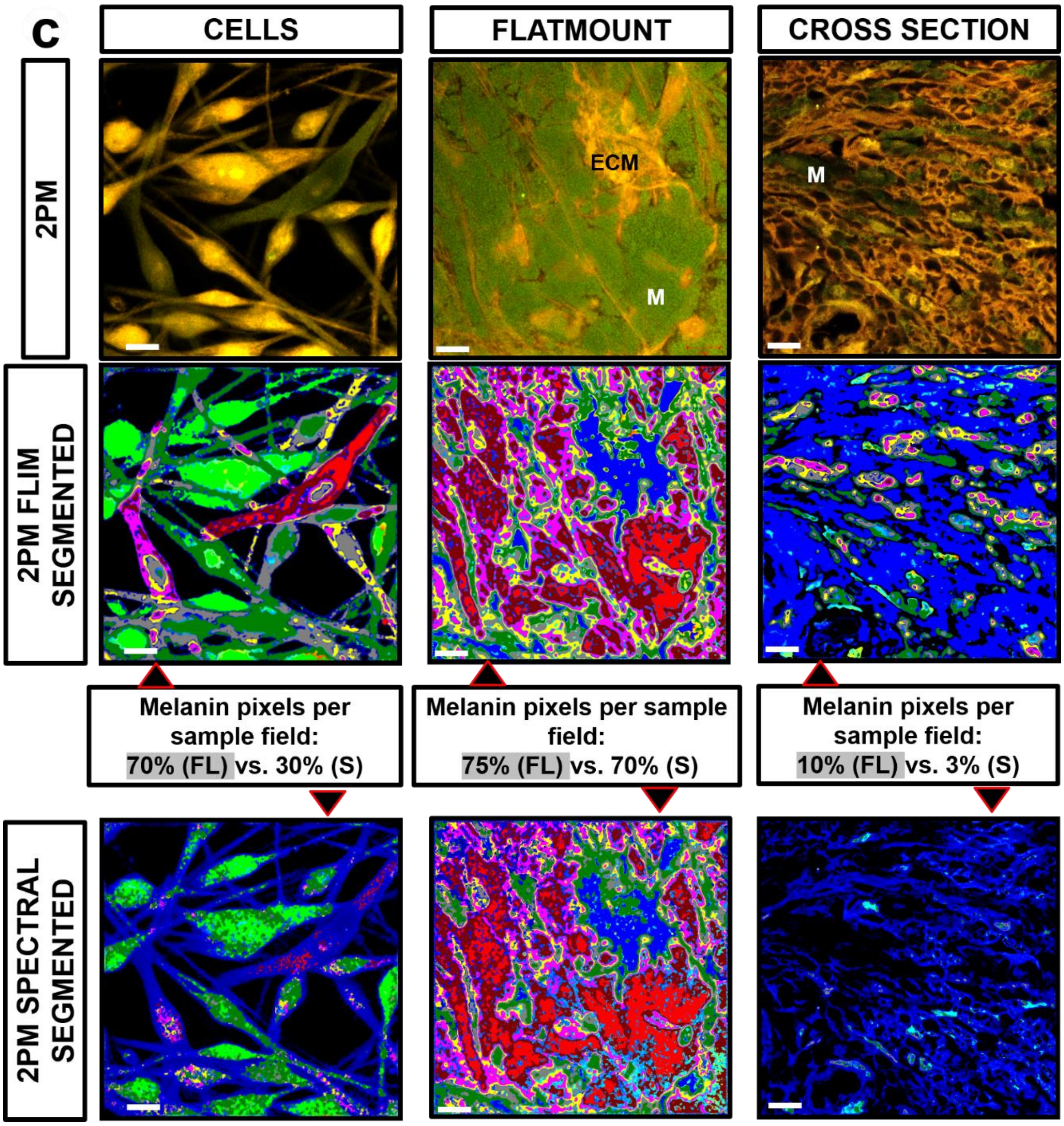

Supplementary Figure 8c: Comparing fraction percentage of pixels representing intracellular melanin per total sample field pixels between segmented FLIM and spectral phasor plot. The non-descanned acquired FLIM data had a higher signal to noise ratio than the spectral emission data. M = HCM, ECM = Extracellular matrix, G = X coordinate of phasor transform (‘real’ unitless phasor component), S = Y coordinate of phasor transform (‘imaginary’ unitless phasor component), Scale bar = 10μm.

Supplementary Note 9

Other non-destructive and non-invasive approaches used to study the intracellular melanin content in skin and hair. Other complementary non-destructive and non-invasive approaches have been used to study the intracellular melanin content in skin and hair [13, 14, 15]. Eumelanin and pheomelanin have distinct photoionization thresholds and vastly different transient absorption responses [5, 16, 17, 18]. A multiphoton technique based on non-linear optical pump-probe spectroscopy was developed, where two ultrafast laser pulses (pump and probe) were used to query the transient excited-state and ground-state photodynamics of the melanins. As a result, microscopic distribution of eumelanin and pheomelanin was directly identified in fixed and label-free human skin pigmented lesions [19, 20]. However, because laser light phase modulation is critical in non-linear optical based imaging [21], there is potential for the two endmembers of a single pump-probe wavelength combination to completely cancel each other [19], affecting the measurement sensitivity. CARS microscopy has been used to examine cytoplasmic melanins; this involves a four-wave non-linear mixing process matching the beat frequency between the interacting pump and stokes beams with the frequency of the Raman active molecular vibration [22]. As a result, the CARS signal of the melanin molecule is enhanced compared to its spontaneous Raman scattering signal [18, 22]. Wang and colleagues (2016) successfully applied this label-free vibrational imaging to directly visualize the distribution of pheomelanin in mouse skin and hair *in vivo* and human skin amelanotic melanoma *in situ* [15]. However, the vibrational peaks from eumelanins were identified within the fingerprint region (FR) of the Raman spectrum (400 cm-1 to 1500 cm-1 frequency range). This made it difficult to isolate the signals from eumelanins because of the spectral interference from other endogenous biochemical species [15]. Taken together, the limitations of the above imaging techniques added gravitas to our decision to apply 2PM FLIM and spectral phasor plot techniques as the preferred approach in this study.

Supplementary Note 10

Inclusion of a red-brown feather, amelanotic human eye melanomas and fetal choroid for melanin controls. The pigmentation of bird plumage is mainly contributed by melanins and carotenoids. Red pigeon feathers have been previously identified to contain pheomelanin [23,

[24, 25], and we propose that red-brown feathers provide a mixed ratio of eumelanin/pheomelanin. Strong pheomelanin signals have been reported for amelanotic human skin melanomas imaged using CARS microscopy [15], and by extrapolation, for amelanotic eye melanomas. Although earlier studies of fetal human eyes reported no detectable dark melanin within the uveal tract at 22 weeks' gestation [26], fetal pre-melanocytes (melanoblasts) do contain both stage II and III melanosomes, and express melanogenesis markers including melanogenic transcription factor microphthalmia-associated transcription factor (MITF) and enzyme tyrosinase related protein 2 (TYRP-2), consistent with these cells being capable of pheomelanogenesis [27, 28].

Supplementary Note 11

Effects of different paraformaldehyde fixation times on fluorescence lifetime and spectral emission characteristics of intracellular melanins. The effects of different paraformaldehyde fixation times on the fluorescence lifetime and spectral emission characteristics of intracellular melanins *in situ* was also investigated. Flatmount tissue with dark pigmented HCMs and synthetic melanin (Sigma-Aldrich) were fixed with 2% paraformaldehyde (Sigma-Aldrich) in PBS (pH 7.4) for 30 minutes, 1 hour, 3.5 hours, 12 hours and 24 hours, followed by PBS rinses (3x; 5 minutes/rinse). The fixed tissue and synthetic melanin were examined for their fluorescence lifetimes and spectral emission characteristics using 2PM FLIM and spectral imaging-phasor plot.

FLIM measurements and λ emission scans on 'very dark' pigmented human choroidal flatmounts and synthetic melanin after 2% paraformaldehyde/PBS fixation for 30 minutes, 1 hour, 3.5 hours, 12 hours and 24 hours. The fluorescence lifetime and spectral emission characteristics of HCM-localized melanin *in situ* and synthetic melanin was not affected by fixation up to 24 hours (**Supplementary Figure 11**). For all fixation periods, phasor clusters mapped to intracellular and synthetic melanin consistently identified the same regions of the phasor plot and their fluorescence emission was detected consistently between 470-690 nm.

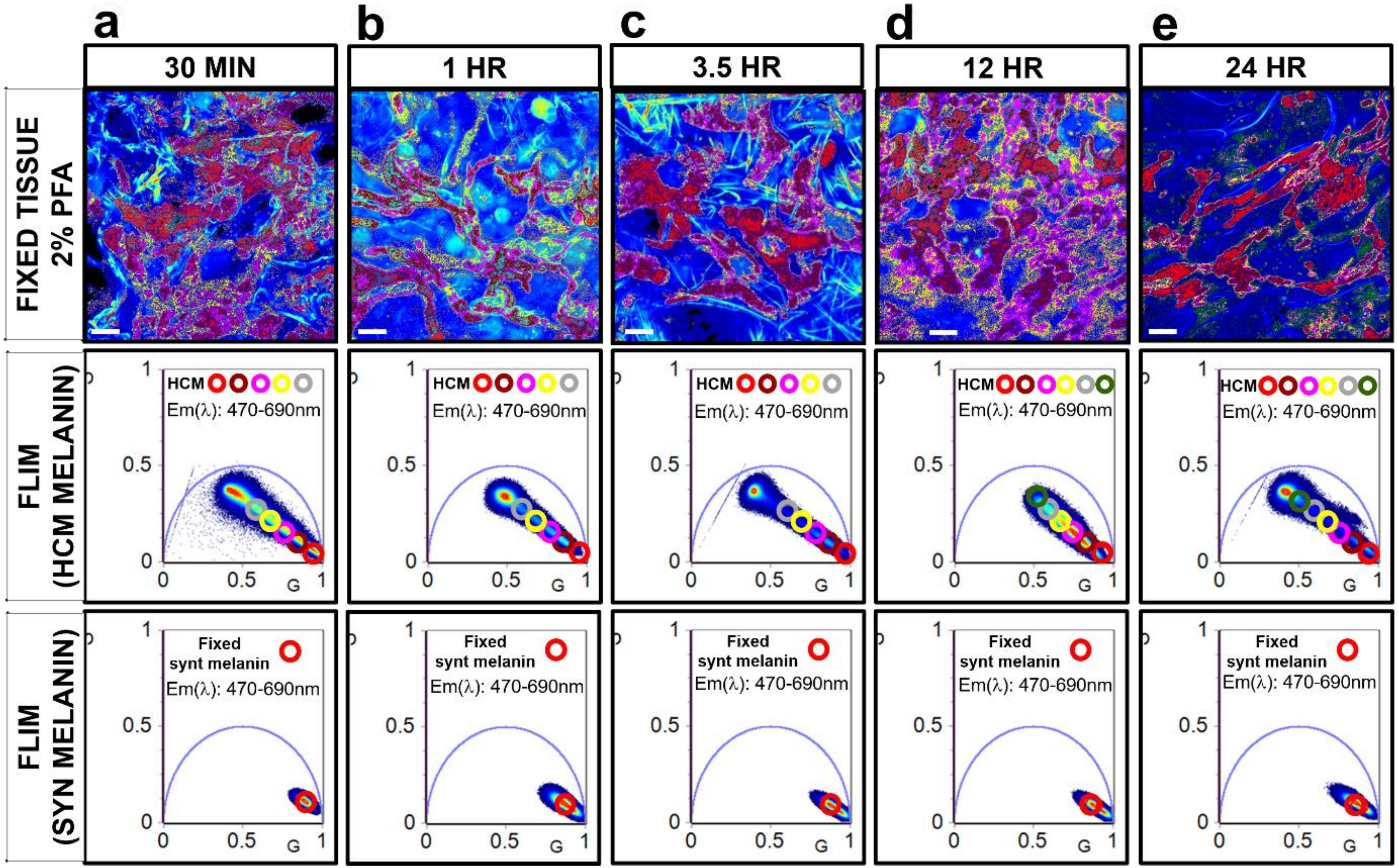
a
b
c
d
e
30 MIN
1 HR
3.5 HR
12 HR
24 HR
FIXED TISSUE 2% PFA
FLIM (HCM MELANIN)
FLIM (SYN MELANIN)
HCM
Em(λ): 470-690nm
Fixed synt melanin
G

Supplementary Figure 11: Effects of fixation on fluorescence lifetimes and spectral emission characteristics of melanins. The 'very dark' pigmented flatmounts and synthetic melanin were fixed at (a) 30 minutes, (b) 1 hour, (c) 3.5 hours, (d) 12 hours and (e) 24 hours, and fluorescence lifetimes and spectral emission were measured. The different fixation times did not affect their fluorescence characteristics. G = X coordinate of phasor transform ('real' unitless phasor component), S = Y coordinate of phasor transform ('imaginary' unitless phasor component), Scale bar = 10μm.

Supplementary Note 12. FLIM and spectral phasor segmentation of all choroidal tissue specimens.

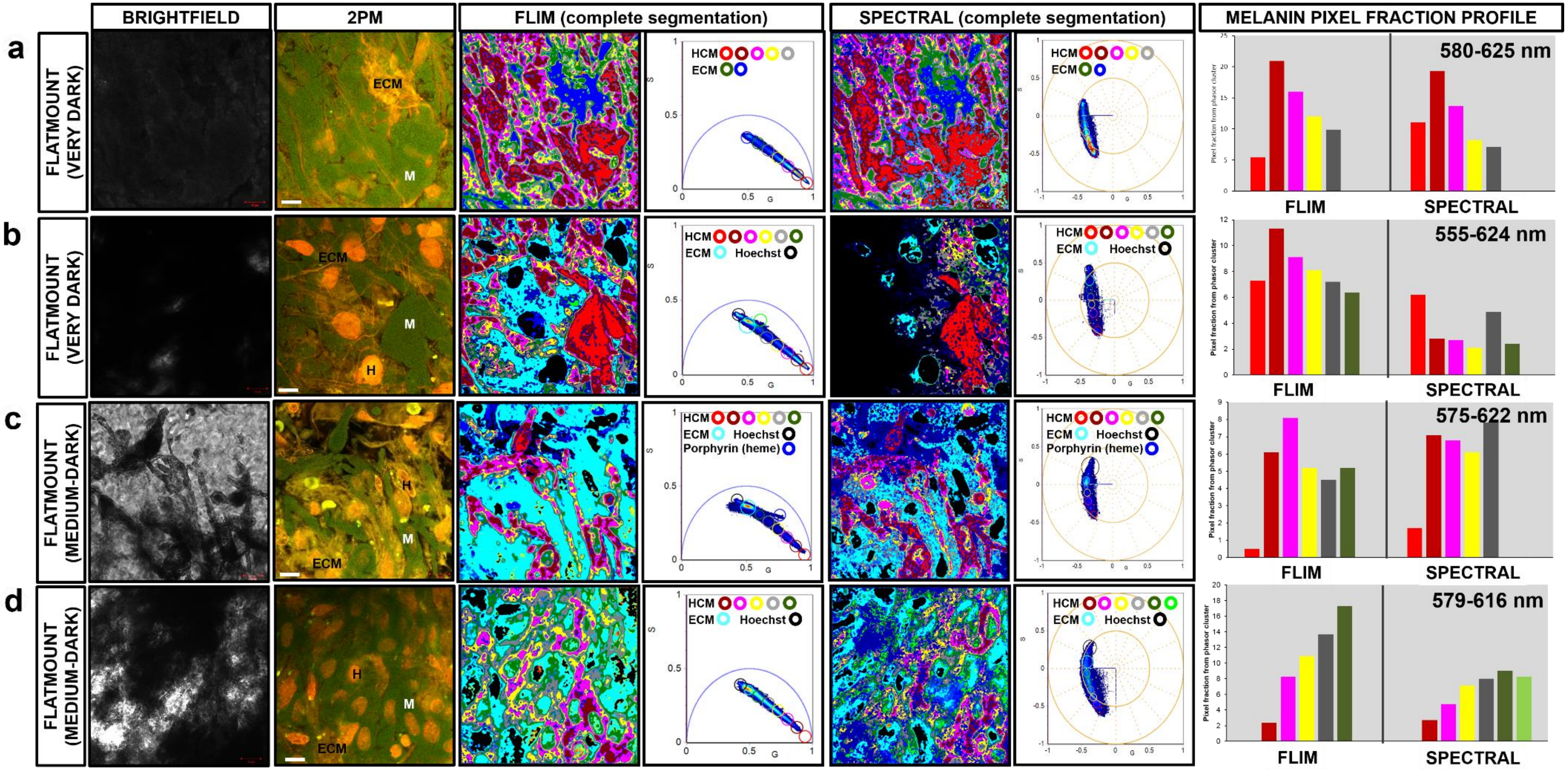

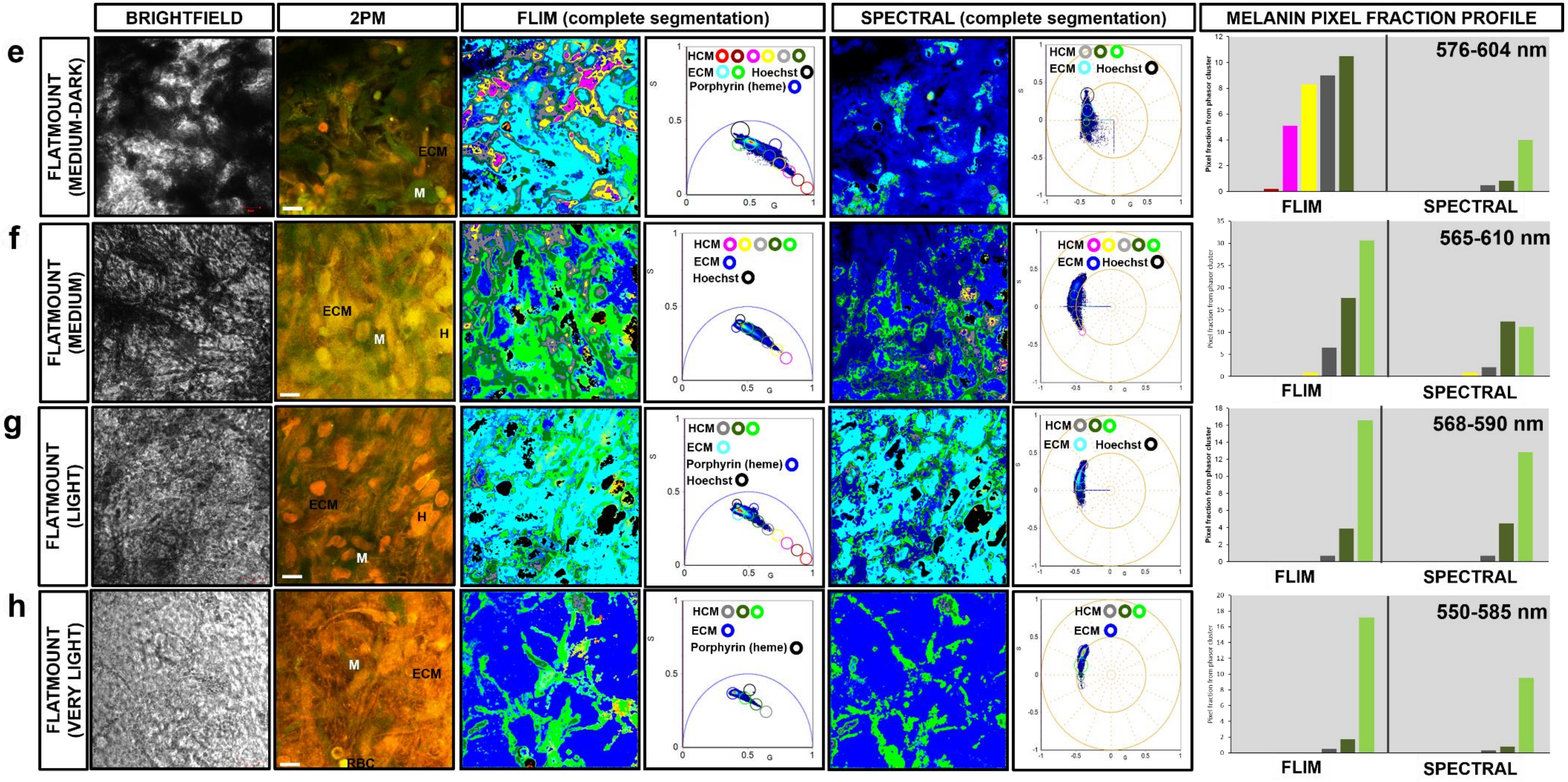
BRIGHTFIELD
2PM
FLIM (complete segmentation)
SPECTRAL (complete segmentation)
MELANIN PIXEL FRACTION PROFILE
e
FLATMOUNT (MEDIUM-DARK)
ECM
M
HCM
ECM
Hoechst
Porphyrin (heme)
576-604 nm
FLIM
SPECTRAL
f
FLATMOUNT (MEDIUM)
ECM
M
H
565-610 nm
g
FLATMOUNT (LIGHT)
ECM
H
M
568-590 nm
h
FLATMOUNT (VERY LIGHT)
M
ECM
RBC
550-585 nm
Pixel fraction from phasor cluster

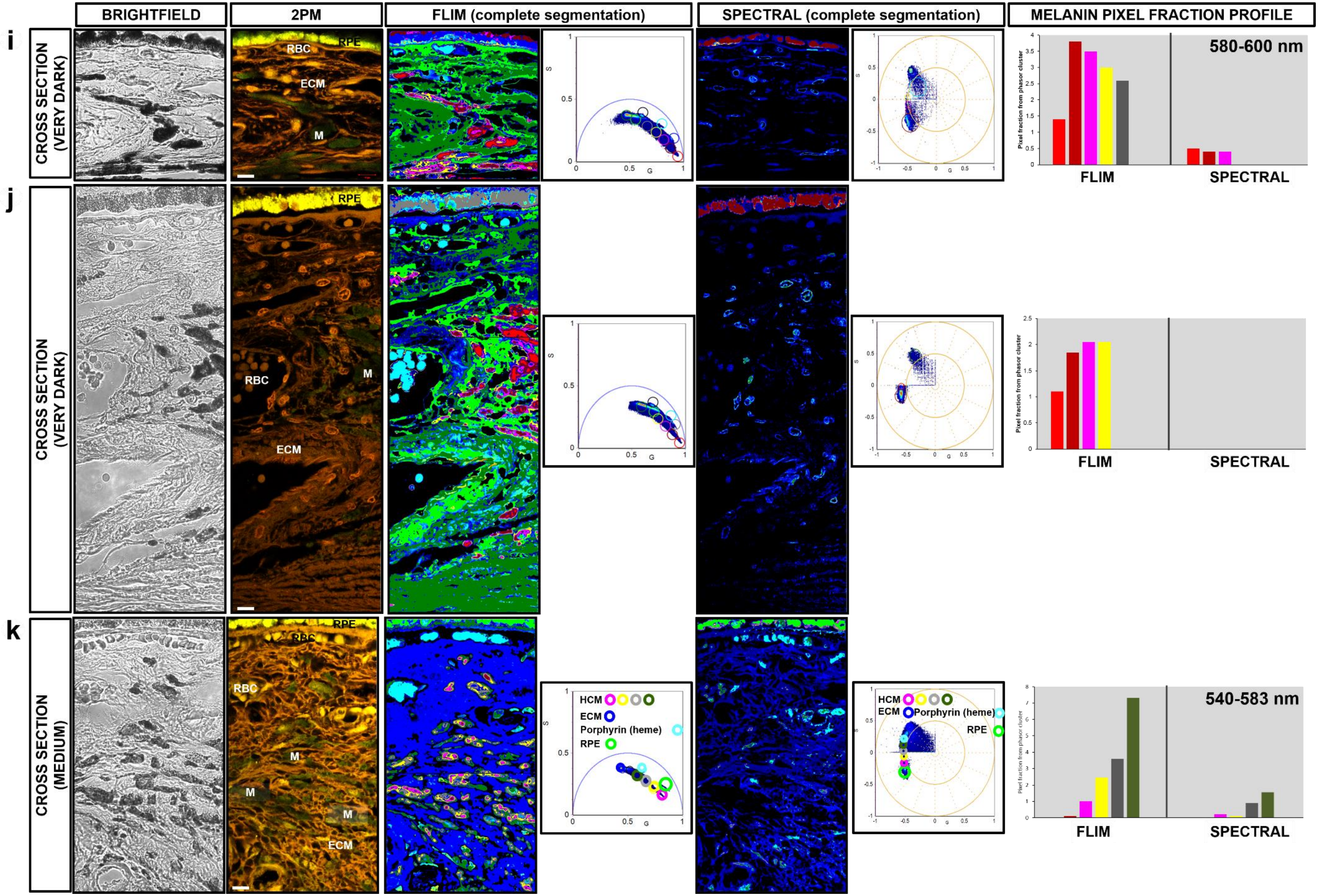

BRIGHTFIELD
2PM
FLIM (complete segmentation)
SPECTRAL (complete segmentation)
MELANIN PIXEL FRACTION PROFILE
i
CROSS SECTION (VERY DARK)
RPE
RBC
ECM
M
580-600 nm
FLIM
SPECTRAL
j
CROSS SECTION (VERY DARK)
RPE
RBC
M
ECM
FLIM
SPECTRAL
k
CROSS SECTION (MEDIUM)
RPE
RBC
M
M
M
ECM
HCM
ECM
Porphyrin (heme)
RPE
540-583 nm
FLIM
SPECTRAL

Supplementary Figure 12: (a-h) Image segmentation of 2PM FLIM and spectral phasor analyses of HCMs in tissue flatmounts and surrounding choroidal structures. (i-k) Image segmentation of 2PM FLIM and spectral phasor analyses of HCMs in tissue cross-sections and surrounding choroidal structures. M = HCM, ECM = Extracellular matrix, H = Hoechst 33342, RPE = Retinal pigment epithelium, RBC = Red blood cell, G = X coordinate of phasor transform (‘real’ unitless phasor component), S = Y coordinate of phasor transform (‘imaginary’ unitless phasor component), Scale bar = 10μm.

References

1. Gurcan MN, Boucheron LE, Can A, Madabhushi A, Rajpoot NM, Yener B. Histopathological image analysis: a review. *IEEE Rev Biomed Eng* **2**, 147-171 (2009).

2. Marcu L, French PMW, Elson DS. *Fluorescence Lifetime Spectroscopy and Imaging: Principles and Applications in Biomedical Diagnostics*. CRC Press (2014).

3. Nighswander-Rempel SP, Riesz J, Gilmore J, Meredith P. A quantum yield map for synthetic eumelanin. *The Journal of chemical physics* **123**, 194901 (2005).

4. Scholz M, Stankovic G, Seewald G, Leupold D. Uncovering of melanin fluorescence in human skin tissue. (2007).

5. Ye T, Yurtsever G, Fischer M, Simon JD, Warren WS. Imaging melanin by two-photon absorption microscopy. (2006).

6. Krasieva TB*, et al.* Two-photon excited fluorescence lifetime imaging and spectroscopy of melanins in vitro and in vivo. *Journal of biomedical optics* **18**, 31107 (2013).

7. Stringari C, Nourse JL, Flanagan LA, Gratton E. Phasor fluorescence lifetime microscopy of free and protein-bound NADH reveals neural stem cell differentiation potential. *PloS one* **7**, e48014 (2012).

8. Zoumi A, Yeh A, Tromberg BJ. Imaging cells and extracellular matrix in vivo by using second-harmonic generation and two-photon excited fluorescence. *Proceedings of the National Academy of Sciences of the United States of America* **99**, 11014-11019 (2002).

9. Perry SW, Burke RM, Brown EB. Two-photon and second harmonic microscopy in clinical and translational cancer research. *Ann Biomed Eng* **40**, 277-291 (2012).

10. Chen J*, et al.* Spectroscopic characterization and microscopic imaging of extracted and in situ cutaneous collagen and elastic tissue components under two-photon excitation. *Skin research and technology : official journal of International Society for Bioengineering and the Skin* **15**, 418-426 (2009).

11. Riesz J, Gilmore J, Meredith P. Quantitative photoluminescence of broad band absorbing melanins: a procedure to correct for inner filter and re-absorption effects. *Spectrochim Acta A Mol Biomol Spectrosc* **61**, 2153-2160 (2005).

12. Malacrida L, Gratton E, Jameson DM. Model-free methods to study membrane environmental probes: a comparison of the spectral phasor and generalized polarization approaches. *Methods Appl Fluoresc* **3**, (2015).

13. Matthews TE*, et al.* In vivo and ex vivo epi-mode pump-probe imaging of melanin and microvasculature. *Biomedical optics express* **2**, 1576 - 1583 (2011).

14. Simpson MJ, Wilson JW, Phipps MA, Robles FE, Selim MA, Warren WS. Nonlinear microscopy of eumelanin and pheomelanin with subcellular resolution. *The Journal of investigative dermatology* **133**, 1822-1826 (2013).

15. Wang H*, et al.* In vivo coherent Raman imaging of the melanomagenesis-associated pigment pheomelanin. *Scientific reports* **6**, 37986 (2016).

16. Samokhvalov A. Heterogeneous Photocatalytic Reactions of Sulfur Aromatic Compounds. *Chemphyschem : a European journal of chemical physics and physical chemistry* **12**, 2870-2885 (2011).

17. Morgan AM, Lo J, Fisher DE. How does pheomelanin synthesis contribute to melanomagenesis?: Two distinct mechanisms could explain the carcinogenicity of pheomelanin synthesis. *Bioessays* **35**, 672-676 (2013).

18. Evans CL. Nonlinear Optical Microscopy for Melanoma: Challenges, Tools, and Opportunities. *Photochem Photobiol*, (2018).

19. Thompson A, Robles FE, Wilson JW, Deb S, Calderbank R, Warren WS. Dual-wavelength pump-probe microscopy analysis of melanin composition. *Scientific reports* **6**, 36871 (2016).

20. Matthews TE, Piletic IR, Selim MA, Simpson MJ, Warren WS. Pump-Probe Imaging Differentiates Melanoma from Melanocytic Nevi. *Science Translational Medicine* **3**, 71ra15-71ra15 (2011).

21. Fischer MC, Wilson JW, Robles FE, Warren WS. Invited Review Article: Pump-probe microscopy. *Rev Sci Instrum* **87**, 031101 (2016).

22. Thiel C. Four-wave mixing and its applications. *Faculty of Washington, Washington DC*, (2008).

23. Haase E, Ito S, Sell A, Wakamatsu K. Melanin concentrations in feathers from wild and domestic pigeons. *J Hered* **83**, 64 – 67 (1992).

24. Ito S, Wakamatsu K. Quantitative Analysis of Eumelanin and Pheomelanin in Humans, Mice, and Other Animals: a Comparative Review. *Pigment cell research / sponsored by the European Society for Pigment Cell Research and the International Pigment Cell Society* **16**, 523 – 531 (2003).

25. Galvan I, Solano F. Bird Integumentary Melanins: Biosynthesis, Forms, Function and Evolution. *Int J Mol Sci* **17**, 520 (2016).

26. Eady RAJ, Gunner DB, Garner A, Rodeck CH. Prenatal Diagnosis of Oculocutaneous Albinism by Electron Microscopy of Fetal Skin. *Journal of Investigative Dermatology* **80**, 210-212 (1983).

27. Cichorek M, Wachulska M, Stasiewicz A, Tymińska A. Skin melanocytes: biology and development. *Advances in Dermatology and Allergology/Postępy Dermatologii I Alergologii* **30**, 30-41 (2013).

28. Wasmeier C, Hume AN, Bolasco G, Seabra MC. Melanosomes at a glance. *Journal of Cell Science* **121**, 3995- 3999 (2008).
